# Decoding visual object recognition from EEG signals

**DOI:** 10.1371/journal.pone.0351872

**Published:** 2026-06-24

**Authors:** Yiwen Kang, Mehdy Dousty, Farnaz Khodami, Ervin Sejdić

**Affiliations:** 1 Department of Electrical and Computer Engineering, University of Toronto, Toronto, Ontario, Canada; 2 Vector Institute for Artificial Intelligence, Toronto, Ontario, Canada; 3 North York General Hospital, Toronto, Ontario, Canada; Aristotle University of Thessaloniki, GREECE

## Abstract

Brain–computer interfaces (BCIs) and clinical EEG require compact and interpretable decoders, yet scalp sensors mix cortical signals and blur frequency-specific activity. Identifying which cortical regions and features carry discriminative visual information enables efficient, anatomically grounded object recognition decoding. This study localizes the cortical sources of informative EEG signals and identifies compact, mechanism-guided features that are most efficient given fixed data or compute budgets. To address this, we construct a source-space decoding pipeline that projects sensor signals onto anatomically defined cortical regions. Trial-wise activity is summarized within regions of interest (ROIs), and four feature families are extracted from each ROI: band-limited power (delta–gamma), line length (LL) for transient activity, temporal morphology, and couplings reflecting coordination between regions. Per-participant Random Forest (RF) classifiers are trained, and generality is quantified as consistency and ROI importance rankings across participants. A low-dimensional representation based on line length yields the strongest overall performance, while temporal morphology and coupling features contribute less under short RSVP (Rapid Serial Visual Presentation) trials. Relative to the EEG-ImageNet sensor-space baseline (310 features), the 24-ROI LL-only stack shows higher reported mean accuracy while using 92% fewer features (24 features), while a finer-grained, extended visual-pathway ROI set shows higher reported mean accuracy while using 84% fewer features (50 features). Adding a small, anatomically constrained high-γ block produces near-tied performance rather than a consistent improvement. These findings indicate that, for single-trial 0.5 s RSVP decoding, most discriminative information is captured by simple time-domain structure in anatomically defined ROIs. High-γ power remains a useful reference feature family, but its incremental value is limited once LL is included. By grounding features in neuro-informed regions, this approach compares favorably, at the level of reported mean accuracy, with the sensor-space baseline while providing clear anatomical attribution at substantially lower dimensionality, supporting lightweight and interpretable EEG decoding.

## Introduction

Visual object recognition engages distributed cortical systems along the ventral and dorsal pathways, with frequency-specific activity supporting their interaction: gamma is linked to feedforward visual processing, while alpha and beta are associated with feedback and control; interregional coupling reflects coordination among these areas [1,2]. Understanding where in the cortex discriminative visual information resides and which neural features encode it, is essential both for advancing neuroscience and for developing practical BCIs that are anatomically interpretable and clinically deployable.

Most EEG-based visual decoding studies operate in sensor space. Engineered features, such as common spatial patterns (CSP) and filter bank CSP (FBCSP) [3], along with Riemannian covariance methods [4] perform strongly but offer limited anatomical attribution and often require many channels and frequency bands. Deep-learning models such as EEGNet can improve decoding performance by learning high-capacity end-to-end representations directly from channel-level signals, but the resulting representations are typically less directly anatomically attributable than compact ROI-level handcrafted features, and their interpretability often depends on post hoc analysis rather than on an explicitly explainable feature space [5,6]. Recent visual-decoding studies draw on large EEG–image datasets that pair high-temporal-resolution EEG with thousands of natural images and tens of thousands of trials per subject, enabling training of high-capacity models for object classification and even image reconstruction or visual-semantic alignment [7–11]. In particular, the EEG-ImageNet benchmark provides recordings from 16 participants viewing 4,000 ImageNet images, with multi-granularity labels (40 coarse and 40 fine categories) and roughly five times more EEG–image pairs than earlier EEG visual benchmarks [12]. Against the focus of existing studies on either sensor-space engineered features or deep end-to-end representations, the question remains open as to how a compact source-space decoder with explicit cortical localization compares within the broader EEG-decoding design space. In particular, this comparison is relevant to the trade-off among accuracy, dimensionality, computational burden, and interpretability.

These model-level trade-offs are compounded by limitations inherent to working in sensor space. Volume conduction mixes multiple cortical generators, obscuring the anatomical locus of discriminative signal [13]. Scalp high-frequency power is particularly susceptible to non-neural contamination: cranial muscle activity inflates power above ~20 Hz, and miniature eye movements can produce broadband transients that mimic gamma, motivating caution in sensor-space interpretations [14–17]. Because frequency-specific mechanisms are region- and pathway-dependent, any claim about the spatial origin of discriminative high-frequency activity should be evaluated in cortical source space rather than sensor-level mixtures. Without such anatomical grounding, it becomes difficult to design features that are anatomically grounded and suitable for portable, clinically transparent systems [18,19].

Source localization addresses these limitations by estimating the cortical origins of scalp-recorded activity and transforming mixed sensor signals into anatomically interpretable, region-specific time series [13]. Recent studies show that projecting EEG to cortical regions of interest as “virtual sensors” can sharpen attribution and enhance decoding performance relative to sensor space; controlled motor-imagery comparisons confirm this with measurable classification gains [20,21]. Practical deployment is supported by real-time toolchains: MNE-CPP enables online source estimation, and MNE-Python supports streaming application of precomputed inverses operators and extraction of ROI time courses for downstream decoding [22–24]. Despite these advances, visual object recognition remains insufficiently characterized in source space. Large EEG–image benchmarks continue to prioritize channel-based representations or deep end-to-end models. This leaves open which cortical regions and low-dimensional feature families are most informative for rapid visual decoding, whether frequency-specific processing claims hold at the source level, and how to design features that are both neurobiologically principled and efficient [8,12].

We build a sensor-to-source decoding pipeline on a standardized benchmark with per-image, time-locked trials [12]. Signals undergo harmonized preprocessing with artifact mitigation and are then projected to the cortex. ROIs are defined using FreeSurfer parcellations at two scales: a compact visual–attentional set and an extended set that increases occipital and frontoparietal coverage [25].

With these source-level ROI signals, we examine three complementary questions about the cortical response using computationally simple features: what changes, via band-limited power; how signals change, via line length and short-horizon morphological features from catch22; and who interacts with whom, via leakage-robust inter-areal coupling. Band power dominates prior pipelines, but cortical specificity in visual tasks is disputed because scalp gamma is vulnerable to ocular and cranial-muscle contamination, motivating source-level evaluations [1,2,26]. Time-domain dynamics are well established clinically, yet remain underexplored for rapid visual decoding. Morphology may contribute beyond power, although evidence is mixed and periodic–aperiodic analyses point to partly independent physiology [27,28]. For connectivity, we include measures that capture complementary forms of coordination: amplitude–amplitude coupling (AAC) tracks cofluctuations in band-limited power, phase–phase coupling (PPC) quantifies phase synchronization, and phase–amplitude coupling (PAC) reflects modulation of high-frequency amplitude by the phase of slower rhythms [29]. PAC, as a cross-frequency interaction, has been proposed as a substrate for large-scale information flow in complex brain networks and is sensitive to both network topology and task- or state-dependent changes [29,30]. However, applying these measures in short windows is challenging because estimates are inconsistent and prone to volume conduction, motivating leakage-robust estimators [31,32]. Mixed conclusions in the literature likely reflect differences in preprocessing, source modeling, window length, and analysis space [33,34]. We therefore compare these feature families under matched preprocessing and source localization. Per-participant RF classifiers are trained with identical pipelines, and generality is summarized as cross-participant consistency of accuracy and feature importance.

Rather than targeting maximal predictive performance, this study examines a different point in the EEG-decoding design space: a compact source-space decoder built from ROI-level handcrafted features with explicit anatomical attribution. The central question is whether rapid visual EEG contains sufficient discriminative information to support accurate decoding under substantially lower feature dimensionality while preserving interpretability regarding the cortical regions and feature families that carry that information. In this sense, recent deep-learning approaches represent a complementary direction, and direct comparison with source-space deep models remains an important avenue for future work.

This work identifies the cortical regions and feature representations that most effectively support rapid visual decoding in the EEG-ImageNet benchmark. We systematically compare low-dimensional feature families, including band-limited power, line length, short-horizon temporal descriptors, and coupling measures (AAC, PPC, PAC). We further establish source-space baselines against sensor-level approaches to assess whether anatomically interpretable representations can recover substantial discriminative information under compact feature budgets, while preserving explicit cortical attribution relative to mixed-sensor baselines.

Our analysis indicates that ROI-level line length provides a strong and compact signature of discriminative information for rapid object decoding, with a consistent cross-participant pattern. A minimal LL-only representation compares favorably, in terms of reported mean accuracy, with the previously reported sensor-space Random Forest reference, while being evaluated under a protocol with explicit separation between training and testing data. The value of this comparison lies not in raw benchmark dominance, but in demonstrating that a lower-dimensional, source-localized, and anatomically interpretable feature stack can recover a large fraction of the decodable information in this paradigm despite practical dataset constraints such as short recording windows and potential redundancy arising from consecutive trial structure. These results further suggest that performance may improve with future datasets incorporating more randomized stimulus presentation, reduced redundancy, and broader trial diversity. It is also effectively tied with variants that add a small, anatomically constrained high-γ block. The corresponding importance profiles are anatomically interpretable at the ROI level. Across label sets, top-ranked ROIs form a distributed posterior–anterior mix spanning early visual, ventral temporal, and frontal regions.

This work advances noninvasive BCI by clarifying which cortical regions and temporal dynamics carry transferable visual information. Identifying high-frequency and transient dynamics along the visual pathway provides concrete design targets for practical systems, enabling efficient decoding with far fewer features than mixed-sensor approaches. For clinical adoption, the anatomical specificity of these representations offers the transparency practitioners require: features map onto established visual structures, decision patterns are auditable, and the lightweight feature set supports deployment on resource-constrained devices. Together, these results translate neuroscientific insight into practical feature design, enabling lean and deployable pipelines for visual communication and control applications.

## Materials and methods

**Fig 1:**
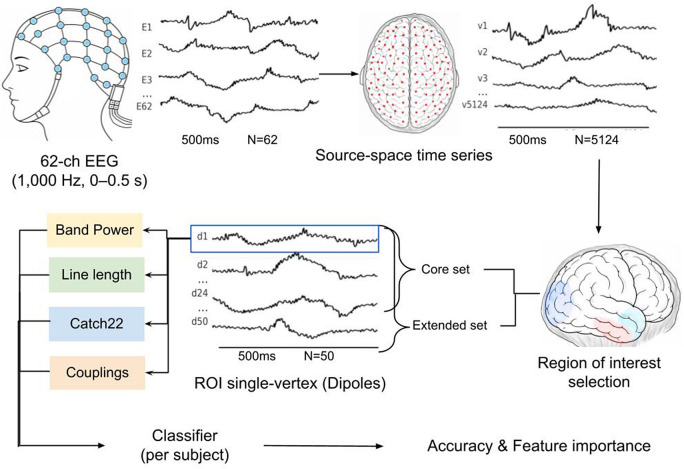
ROI-anchored source-space EEG decoding pipeline. EEG signals were recorded at the sensor level, projected to source space, summarized as ROI single-vertex time series, and used for feature extraction and per-subject classification. Selected schematic elements were redrawn and modified from public-domain (CC0 1.0) source material, including Wikimedia Commons resources.

Fig 1 provides an overview of the ROI-anchored source-space decoding pipeline, comprising preprocessing and artifact mitigation, source modeling with anatomically defined ROIs, feature extraction, and per-participant classification.

### Data and preprocessing

EEG-ImageNet includes recordings from 16 participants, each viewing ∼4,000 images spanning 80 categories with both coarse and fine labels. Coarse labels correspond to 40 visually distinct ImageNet categories, whereas fine labels comprise 40 subordinate categories grouped into five sets of eight visually similar classes that share the same WordNet parent (e.g., musical instruments such as accordion, cello, flute, oboe, snare drum, and trombone) [12]. The released dataset provides 62-channel EEG sampled at 1,000 Hz and segmented into 0.5 s epochs time-locked to stimulus onset [12].

Preprocessing follows a standard EEG denoising pipeline: band-pass filtering (0.5–150 Hz), notch filtering at line noise and harmonics, ICA-based removal of ocular and craniofacial EMG components, common-average re-referencing, and spherical-spline interpolation of bad channels [35]. Bad channels and contaminated epochs are rejected using fixed amplitude/correlation thresholds and muscle-band power criteria. ICA components are labeled using ICLabel [35]. Full preprocessing and classifier hyperparameter settings are provided in S1 Appendix.

### Source modeling and regions of interest

#### Source space.

We perform EEG source localization in MNE [36] using the FreeSurfer fsaverage template anatomy [25] and a three-layer boundary element (BEM) head model. Cleaned 10–20 sensor positions are coregistered to fsaverage, and a cortical surface source space is constructed via icosahedral tessellation with dipoles constrained normal to the cortex [37]. The forward (lead-field) operator is computed from the BEM solution [38]. Noise covariance is estimated using cross-validated shrinkage and applied for whitening [39]. We use depth-weighted L2 minimum-norm with dSPM (Dynamic Statistical Parametric Mapping) noise normalization to obtain per-trial (0–0.5 s post-stimulus) source time series at fixed vertices [40]. These trial-wise vertex signals are summarized per ROI by selecting the vertex nearest the ROI centroid, yielding one ROI time series per hemisphere.

#### ROI definition.

We summarize source time series within anatomically defined cortical regions using FreeSurfer parcellations [41]. The ROIs are chosen to cover the main stages of the human object-vision system while keeping the representation compact and interpretable.

We define two ROI sets: a 24-ROI core parcellation used for the primary analyses, and a 50-ROI extended parcellation used for higher-resolution follow-up analyses.

For the primary analysis (24-ROI core parcellation), 12 regions per hemisphere (24 total) are selected to cover four functional subsystems central to rapid object vision. Early and lateral occipital cortex ROIs capture retinotopic visual field maps and shape-selective processing in occipital and occipito-temporal cortex [42,43]. Ventral temporal ROIs (fusiform and inferior temporal gyri) index category-selective object representations along the ventral stream. Dorsal parietal regions (superior parietal and intraparietal sulcus) and frontal control regions (middle/inferior frontal gyri and precentral cortex) sample nodes of the dorsal attention network involved in spatial selection, evidence accumulation, and goal-directed control [42,44]. Together, these four subsystems provide a concise but mechanistically grounded summary of ventral and dorsal visual pathways and their top–down modulation, which are consistently implicated in object recognition and attentional readout.

For finer anatomical attribution and robustness checks, an extended set of 25 regions per hemisphere (50 total) is defined using Destrieux subdivisions. This set increases coverage of lateral and ventral occipital cortex that better approximate intermediate visual field maps (V3/V4) and motion-sensitive complexes (dorsal occipito-temporal regions), enabling more granular localization within occipital and occipito-temporal cortex [43]. The intraparietal sulcus is split into anterior and posterior segments to capture functional gradients along the dorsal stream. Additional frontal eye field and posterior superior temporal sulcus ROIs are included to better reflect oculomotor control and ventral–lateral contributions related to eye movements, motion, and dynamic object interactions [44,45]. This extended parcellation allows assessment of whether discriminative signals are confined to broad visual and attentional territories or instead depends on more fine-grained subdivisions within these networks. Exact ROI names and atlas label strings appear in S1 Table.

### ROI time-series extraction

ROIs are defined on fsaverage using the Destrieux atlas for the 24-ROI core set and the 50-ROI extended set. For every ROI–hemisphere pair, we select one representative cortical dipole (surface vertex) and extract its trial-wise source time series for feature computation.

Let $V_{r,h}=\{{𝐩}_{j}{\}}_{j=1}^{N_{r,h}}$ denote the MNE surface coordinates (mm) of all vertices belonging to ROI *r* in hemisphere h∈{lh,rh}, obtained from the atlas labels. A common approach is to use the source activity at the solution point nearest the geometric center (centroid) of each ROI as its representative time series [46]. We compute the ROI center as:


$$\begin{array}{c} {𝐜}_{r,h}=\frac{1}{N_{r,h}}\sum_{j=1}^{N_{r,h}}{𝐩}_{j}. \end{array}$$ (1)

We choose the representative vertex as the one closest to the ROI centroid, measured by Euclidean distance, within the same hemisphere:


$$\begin{array}{c} j^{⋆}=\text{arg}\min_{j\in \{1,\ldots ,N_{r,h}\}}{\left\|{𝐩}_{j}-{𝐜}_{r,h}\right\|}_{2}. \end{array}$$ (2)

Given the per-trial source estimate 𝐬(t) at fixed cortical vertices (dSPM-normalized MNE units) on the cortical mesh, the ROI time series is defined as $x_{r,h}(t)=s_{j^{⋆}}(t)$. This single-vertex summary keeps the feature budget small and avoids additional spatial mixing across sulcal boundaries, while hemisphere identity is preserved by performing the selection separately for left and right labels.

As a deliberate design choice, each ROI was summarized by a single representative dipole waveform rather than by within-ROI averaging. In the present pipeline, this representative waveform was taken from the selected source point nearest the ROI centroid, so that each parcel contributed one anatomically localized time series with a fixed and transparent mapping to the downstream feature space. This choice was made to preserve a compact one-feature-per-ROI representation, maintain direct anatomical attribution, and avoid additional cancellation or spatial mixing that can arise when source estimates with heterogeneous local orientations are averaged within a parcel. Template-based EEG source-analysis pipelines commonly perform group-level analyses on anatomically defined ROIs even when individual anatomy and digitized sensor locations are unavailable [47]. In the broader ROI-summarization literature, centroid-based representative waveforms are an established low-dimensional alternative to parcel averaging and PCA/SVD-based summaries [48]. Because the main goal of the present study was to evaluate compact, interpretable ROI-level feature representations rather than to maximize within-ROI variance capture, we adopted this single-dipole summary rule throughout all experiments.

### Feature extraction

Unless otherwise noted, all feature families were computed on the full 0–0.5 s post-stimulus epoch.

#### Band power.

For each ROI time series, we compute log power in canonical frequency bands δ (1–4 Hz), θ (4–8 Hz), α (8–13 Hz), β (13–30 Hz), and γ (evaluated as 30–120 and 70–150 Hz) using Welch’s method with overlapping windows. Welch power was computed with nperseg = min(length,256) and noverlap = nperseg/2. Features are standardized using statistics computed on the training set [49]. This representation captures rhythmic components implicated in visual processing and communication (alpha–beta feedback, gamma feedforward) [2] and enables explicit evaluation of gamma-band contribution.

We test three band-power configurations. We include the five-band δ−θ−α−β−γ stack as a conventional full-spectrum baseline widely used in EEG–BCI systems and relevant to RSVP/P300-related low-frequency activity [50,51]. To isolate the mechanistic distinction between narrowband γ rhythms and broadband high-frequency activity reported in intracranial studies, we additionally test two single-band variants: γ (30–120 Hz) and high-γ (70–150 Hz) [52,53]. We do not train separate δ-, θ-, α-, or β-only models, because low-frequency content is already included in the five-band stack and these bands primarily contribute jointly to slow event-related potential (ERP) complexes.

#### Line length.

Line length provides a time-domain “roughness” measure that is sensitive to fast, broadband transients and aperiodic changes. Given a discrete ROI time series *x*[*n*] over an analysis window of *N* samples, line length is computed as the sum of absolute successive differences [54,55]:


$$\begin{array}{c} LL(x)=\sum_{n=1}^{N-1}\left|x[n+1]-x[n]\right|. \end{array}$$ (3)

Because it accumulates sample-to-sample changes, line length increases with both signal magnitude and rapid fluctuations, making it sensitive to both amplitude and frequency content [56].

It is widely used in clinical EEG and iEEG detection tasks, where it offers complementary information to narrowband power [54]. The inclusion of a broadband-sensitive descriptor is motivated by evidence that high-frequency or broadband power co-varies with local spiking and can serve as a practical proxy for population activity [53], as well as by the need to disentangle periodic from aperiodic components [28].

#### Temporal descriptors (catch22).

For each ROI time series, we compute the 22 canonical time-series statistics from the catch22 library [27]. These features provide lightweight, task-agnostic summaries of short-lag autocorrelation, successive differences, forecastability, entropy, and motif structure, capturing temporal morphology that is not well described by band power alone [27]. Recent work has applied catch22 to neural time series, including EEG dynamics during variable-intensity cycling, sleep and dream mentation, and seizure-related EEG analyses [57–59], supporting its use as a streamlined and transparent descriptor family under tight feature budgets.

We additionally apply principal component analysis (PCA) to the catch22-derived feature matrix to obtain a lower-dimensional representation [60–62]. For each participant, PCA is fit on the training data, and a fixed number of leading components is retained to match the dimensionality of the other feature sets. For the PCA-reduced catch22 representation, PCA is fit separately within each training fold and then applied to the corresponding held-out fold, preventing dimensionality-reduction leakage across cross-validation splits. The target dimensionality is 24 components; when this is infeasible because of sample-count or feature-count constraints, the number of retained components is reduced automatically to the largest valid value. Before PCA, NaN or infinite catch22 entries are replaced by column-wise means. No whitening is applied.

#### Coupling measures.

To capture coordination beyond local power and morphology, we evaluate three coupling descriptors that represent complementary and commonly used modes of interaction in electrophysiology: phase synchrony, cross-frequency phase–amplitude coupling, and envelope cofluctuations [63–65]. Restricting the coupling screen to these canonical forms keeps the representation compact under the short-window RSVP design while still testing distinct coordination mechanisms.

Inter-ROI phase synchrony is computed in 30–120 Hz using the phase-locking value (PLV) [63]. Within each ROI, PAC is quantified using the modulation index (MI), with α-band phase (8–12 Hz) and high-γ amplitude (70–150 Hz) [64,66,67]. AAC is computed as the Pearson correlation between band-limited envelopes in the β (13–30 Hz) and high-γ (70–150 Hz) bands; to mitigate source-leakage effects, envelopes are extracted after symmetric multivariate orthogonalization of the ROI time series [32].

We evaluate each coupling family separately and also form an all-couplings representation by concatenating PLV, PAC, and AAC. For each family, we include two high-SNR variants: an amplitude-weighted (AW) variant that weights each trial’s coupling estimate by the mean high-γ amplitude of the involved ROI(s) [65,66], and a threshold-gated (TG) variant computed only on samples where high-γ amplitude exceeds the 75th percentile of its trial-wise distribution, analogous to burst-restricted analyses in electrophysiology [68,69].

### Tasks and evaluation protocol

We follow the three official EEG-ImageNet setups: *All-80* (all 80 categories), *Coarse-40* (40 coarse labels), and *Fine-40* (the average performance over five predefined 8-class groups) [12].

Before running the official splits, we performed all hyperparameter tuning on a visually distinctive, class-balanced 8-class subset (African elephant, airliner, banana, electric guitar, folding chair, desktop computer, lycaenid, revolver). These categories span diverse object types (animals, vehicles, tools, food) and minimize fine-grained visual similarity. Coarse object-category distinctions are known to be robustly represented in early visual EEG responses during rapid viewing paradigms [70]. Accordingly, feature families and hyperparameters that do not achieve reliable performance on this subset are unlikely to perform better on the harder official fine- and coarse-grained benchmarks. Using an easier but representative subset also keeps the pilot search computationally tractable and avoids holding out entire participants for validation. This 8-class pilot is not used as an official benchmark; it serves solely to select feature families, ROI granularity, and hyperparameters that are then fixed for All-80, Coarse-40, and Fine-40.

To enable direct comparison with EEG-ImageNet baselines, we adopt a per-subject training scheme. The same classifier specification and fixed feature stack are used for every participant, with models trained separately on each participant’s data. No cross-subject training is performed, so results reflect within-subject decoding. Within each participant and label set, we use stratified 5-fold cross-validation: trials are partitioned into five folds with matched class proportions, models are trained on four folds and evaluated on the held-out fold, and accuracies are averaged across folds to obtain one score per participant. Hyperparameters and the feature stack are fixed a priori from the exploratory 8-class pilot and held constant for All-80, Coarse-40, and Fine-40.

### Staged feature-family evaluation and selection

Exhaustive search over all feature subsets is infeasible for moderate- to high-dimensional EEG representations and is particularly ill-suited to datasets with relatively few participants and trials. In this regime, the combinatorial growth of candidate subsets exacerbates the curse of dimensionality and leads to overfitting, even under strong regularization. We therefore adopt a staged feature-selection strategy [71,72] that is conceptually similar to hybrid filter–wrapper methods proposed for EEG [73,74]. In such hybrid schemes, an initial filter stage uses simple performance criteria to discard clearly suboptimal candidates (for example, comparing decoding accuracy under a fixed classifier and favoring more compact representations), while a subsequent wrapper stage evaluates a much smaller set of feature stacks with the full classifier. This two-stage design reduces the search space while still allowing interaction effects between features to be captured in the wrapper stage.

In our case, the standalone 8-class evaluation of each feature family acts as the filter-like stage: entire families (band power, line length, temporal descriptors, couplings) and their internal configurations are screened, and only the strongest configurations are retained as baselines or candidate add-ons. The subsequent baseline-plus-feature-add-on experiments at 24- and 50-ROI resolution then play the role of the wrapper stage, in which the full classifier is trained on a small number of compact feature stacks and their performance is compared after aggregation across subjects.

We first evaluate each feature family on the 8-class pilot subset using the 24-ROI core parcellation. Band powers, line length, catch22 temporal descriptors and coupling features are each assembled into a family-specific representation, trained using the same classifier, and compared in terms of accuracy aggregated across participants (mean ± standard deviation (SD)).

From the band power configurations, we retain a single frequency-domain power representation with adequate decoding accuracy and stable cross-subject performance as the primary baseline, because band-limited power remains among the most widely used feature types in EEG-based BCI systems [75,76]. If a feature family outside the band power group attains comparable or higher standalone performance on the same pilot task, that family is also retained as a second baseline. Subsequent feature-addition analyses are then run separately on top of each baseline, allowing direct comparison between improvements over a conventional spectral descriptor and improvements over the best-performing representation identified in our data.

Candidate feature add-ons are then defined in a constrained, data-guided manner. Rather than exhaustively recombining all feature types, we use the single-family screening results and per-feature importance profiles to identify subsets or variants of each family that appear informative and complementary. These candidate stacks are subsequently evaluated on the 8-class pilot task at both spatial resolutions (24-ROI core and 50-ROI extended parcellations) as additions to each baseline.

Configurations that provide the most reliable improvement over their respective baselines while remaining compact are retained as the final feature stacks for each baseline (the spectral band power baseline and the selected non-spectral baseline) and each ROI resolution (24-ROI core and 50-ROI extended). These stacks are then applied unchanged to the official All-80, Coarse-40, and Fine-40 splits, where separate models are trained per participant and performance is summarized as the cross-subject mean ± SD accuracy across participants.

### Classifiers

In this study we focus on five standard supervised classifiers commonly used in EEG decoding: linear SVM, ridge classifier, KNN, $\ell_{2}$-regularized logistic regression, and RF. Linear models are widely adopted in EEG-based BCI pipelines as strong baselines on band power and related features and provide simple linear decision boundaries [75]. Ridge classification provides an additional linear baseline with $\ell_{2}$ shrinkage, while KNN offers a simple distance-based nonparametric classifier after feature standardization. In contrast, RF can capture nonlinear interactions among heterogeneous features, including spectral, temporal, and connectivity measures. It is relatively robust to differences in feature scale and monotonic transformations, and provides model-based feature importance estimates that can be used to inspect and summarize learned feature contributions.

To select a single classifier for the main analyses, all five models are evaluated on the same 8-class pilot subset using identical data partitions and the same feature configuration. Classifier comparison is performed using the 24-ROI high-γ band power representation as a representative spectral test set. Band-limited power features, and in particular visually driven γ/high-γ activity, are among the most widely used descriptors in EEG-based decoding and BCI systems and have well-established links to local visual population activity [52,75]. The classifier yielding the best trade-off between cross-subject mean accuracy, stability across folds, and implementation simplicity is then fixed and used for all subsequent experiments. This staged design allows the official label-set analyses to focus on differences in feature representation and anatomical attribution under a fixed downstream classifier, rather than treating the final benchmark stage as an open-ended search for the best predictive model. In this sense, the primary objective of the study is not exhaustive classifier optimization, but a controlled comparison of compact source-space feature families under a shared decoding framework. The same stratified 5-fold partitions are used for all classifiers and feature configurations on both the pilot and the official label sets. Full hyperparameter settings are listed in S1 Appendix.

Because our downstream analyses rely on feature-level attribution, we require a well-defined notion of feature importance for each candidate classifier. For every participant and classifier, we use a model-agnostic permutation procedure to quantify feature contributions. After fitting the model, we first compute a reference accuracy on the corresponding evaluation split using the unpermuted features [77]. We then permute the values of each feature across trials, one at a time, and recompute accuracy; the resulting drop relative to the reference value is taken as that feature’s importance score, with larger drops indicating greater contribution to decoding. This permutation-based importance is applied uniformly to RF, linear SVM, ridge classifier, KNN, and $\ell_{2}$-regularized logistic regression under identical data partitions and feature configurations.

For linear SVM, ridge classifier, and $\ell_{2}$-regularized logistic regression, we additionally compute a coefficient-based importance proxy: features are *z*-scored before training, and the absolute values of the learned weights (aggregated across classes via the $\ell_{2}$ norm) are used as secondary per-feature importance scores. For KNN, no analogous coefficient-based proxy is available; feature attribution for this model is therefore limited to the same model-agnostic permutation procedure described above. All anatomical and representation-level summaries reported in this work are based on permutation-importance scores: for each feature, importance values are averaged across participants (mean ± SD) and then pooled by ROI or by feature family to quantify anatomical attribution (ROI-level contributions) and representation attribution (feature-family contributions).

## Results

Results are organized in four stages. First, five candidate classifiers (linear SVM, ridge classifier, KNN, $\ell_{2}$-regularized logistic regression, and RF) are compared on the 8-class pilot using 24-ROI high-γ band power, and a single model is selected for subsequent analyses. Second, with this classifier fixed, feature families are evaluated in isolation on the same 8-class 24-ROI pilot to characterize standalone decoding performance. Third, we retain at most one spectral and one non-spectral baseline from this screen and assess compact baseline-plus-add-on stacks on the 8-class pilot at both ROI resolutions (24-ROI core and 50-ROI extended). Finally, the selected stacks are applied unchanged to the full EEG-ImageNet benchmark (All-80, Coarse-40, Fine-40) at both ROI resolutions, and cross-subject performance is summarized.

### Classifier and feature-family screening

#### Classifier selection.

On the 8-class pilot using the 24-ROI high-γ band power representation, the five candidate classifiers showed broadly similar cross-subject decoding performance overall (Table 1). Linear SVM, ridge classifier, KNN, and $\ell_{2}$-regularized logistic regression reached 0.650±0.106, 0.627±0.102, 0.642±0.116, and 0.659±0.108 accuracy (mean ± SD across 16 subjects), respectively, whereas RF achieved 0.674±0.094. Taken together, RF provided the highest mean accuracy with slightly lower variability across participants. Combined with its ability to capture nonlinear interactions among heterogeneous features and to integrate seamlessly with our permutation-based feature-importance analysis, we adopt RF as the classifier for all subsequent experiments.

**Table 1:** Classifier comparison accuracy on the 8-class pilot using 24-ROI high-γ band power representation. Values are mean ± SD across 16 subjects.

Classifier	Accuracy
Linear SVM	0.650±0.106
Ridge classifier	0.627±0.102
KNN	0.642±0.116
$\ell_{2}$ Logistic Reg.	0.659±0.108
Random Forest	0.674±0.094

#### Band power.

High-γ power achieved the highest mean accuracy (0.674±0.100), exceeding γ (30–120 Hz) (0.630±0.110; + 4.4%) and the five-band configuration (0.623±0.105; + 5.1%) despite using 5× fewer features than the latter (Table 2). Compared with γ (30–120 Hz) alone, high-γ showed a modest but consistent accuracy gain and slightly reduced across-subject variance. In the five-band comparison (δ,θ,α,β,γ), the Top-20 features aggregated across participants are all γ-band entries (S1 Fig). Within the γ range, frequency sub-bands show spatial dissociation: high-γ features concentrate in early and ventral visual cortex (V1/V2, fusiform gyrus, inferior temporal cortex), whereas γ (30–120 Hz) retains more frontal and associative entries (S2 and S3 Figs). This pattern suggests that lower-γ components capture additional cognitive or control-related processes beyond sensory encoding.

**Table 2:** 8-class, 24-ROI band power accuracies. Values are mean ± SD across 16 participants; one feature per ROI.

Configuration	No. of Features	Accuracy
High-γ (70–150 Hz)	24	0.674±0.094
γ (30–120 Hz)	24	0.630±0.110
Five-band (δ⋯γ)	120	0.623±0.105

#### Catch22 features.

We evaluate catch22’s 22 canonical time-series descriptors across all 24 ROIs, yielding 528 features per trial to capture temporal morphology (Table 3).

**Table 3:** 8-class, 24-ROI *catch22* accuracies. Values are mean ± SD across 16 participants.

Configuration	Features	Accuracy
catch22 (22 stats × 24 ROIs)	528	0.521±0.096
catch22-PCA (24 PCs)	24	0.394±0.082

The Top-20 catch22 features (S4 Fig) are systematically enriched for short-horizon statistics recurring across multiple ROIs. The most frequent entries include local forecast error (FC_LocalSimple_mean3_stderr), autocorrelation decay time (CO_f1ecac), successive-difference variability (MD_hrv_classic_pnn40), and three-point motif asymmetries (SB_MotifThree_quantile_hh). These descriptors capture transient waveform morphology and local temporal structure rather than sustained oscillatory power, providing complementary, largely non-redundant information relative to frequency-based features [50,70]. The Top-20 features repeatedly include early and ventral visual cortices (V1/V2/IT/fusiform) alongside frontoparietal hubs (dlPFC, superior frontal, IPL/SPL, precuneus, parahippocampal), indicating that discriminative temporal dynamics are distributed across both sensory and control regions. Using catch22 alone yields an accuracy of 0.521±0.096 across 16 subjects, capturing transient waveform morphology and brief temporal irregularities. As such, catch22 serves as a useful supplement to frequency-based approaches by characterizing non-oscillatory signal properties that power spectra may miss.

To test whether this morphology can be compressed into a 24-dimensional linear subspace, we apply PCA to the catch22 feature matrix and retain the first 24 components per participant. Classification using this 24-dimensional PCA representation reduces accuracy to 0.394±0.082, corresponding to an absolute drop of approximately 0.13 absolute (about 24% relative) compared with the full catch22 representation (0.521±0.096).

#### Line length.

In 0.5 s RSVP epochs, where a dominant signal takes the form of broadband bursts in visual cortex, line length provides an effective discriminant. On the 8-class, 24-ROI pilot, LL alone attains an accuracy of 0.747±0.092 across 16 participants. The Top-20 LL features (S5 Fig) are dominated by early and ventral visual cortex (V1/V2, fusiform, inferior temporal) with additional contributions from dorsal parietal and medial hubs (superior parietal lobule, precuneus). This concentration in visual ROIs indicates that LL primarily captures local, stimulus-locked waveform structure rather than long-range coordination.

#### Coupling features.

Coupling families are evaluated using the three variants: plain, amplitude-weighted, and threshold-gated. Phase synchrony in the high-γ band achieves moderate standalone accuracy (Table 4): plain PLV reaches 0.426±0.078, and amplitude weighting improves this to 0.483±0.087. In contrast, within-ROI PAC and AAC configurations yield low accuracies close to chance (PAC: 0.154±0.030; AAC: 0.166±0.034), and concatenating all three families produces only modest performance gains (all couplings, plain: 0.430±0.085; AW: 0.476±0.089). Threshold gating consistently reduces accuracy across families, indicating that discarding low-amplitude samples in 0.5 s windows inflates estimator variance rather than isolating more informative segments.

**Table 4:** 8-class, 24-ROI coupling accuracies. Values are mean ± SD across 16 subjects. Plain, amplitude-weighted, and threshold-gated variants are compared for each family.

Configuration	No. of Features	Accuracy
PLV (plain, γ 30–120, inter-ROI)	276	0.426±0.078
PLV (AW)	~276	0.483±0.087
PLV (TG)	~276	0.391±0.083
PAC (plain, α→high-γ, within-ROI)	24	0.154±0.030
PAC (AW)	~24	0.154±0.030
PAC (TG)	~24	0.144±0.021
AAC (plain, β↔high-γ, within-ROI)	24	0.166±0.034
All couplings (plain: PLV + PAC + AAC)	324	0.430±0.085
All couplings (AW)	~324	0.476±0.089
All couplings (TG)	~324	0.387±0.077

Permutation-based feature importances confirm that the most informative coupling features are dominated by inter-ROI phase-synchrony edges. For PLV and the concatenated all-couplings stack (AW variants; S6 and S7 Figs), the Top-20 features repeatedly link early and ventral visual ROIs (V1/V2, fusiform, inferior temporal) with dorsal parietal and medial hubs (superior parietal lobule, precuneus, dorsolateral prefrontal cortex, posterior cingulate), consistent with coordinated activity across visual and control networks during RSVP viewing. In contrast, PAC-only and AAC-only maps (S8 and S9 Figs) are flatter and more diffuse, with many ROIs contributing small, similar importance values and few standout entries, in line with the low standalone accuracies obtained from single-trial 0.5 s estimates.

### Baseline selection and feature add-ons

The 8-class pilot serves as an internal feature-family selection stage for the subsequent combination analyses. In line with the staged procedure, we retain at most one spectral and one non-spectral baseline: a single band-power configuration with the highest cross-subject accuracy (mean ± SD across participants) and stability, and any non-spectral family that matches or exceeds this spectral performance.

Within the band-power family, the 70–150 Hz high-γ representation yields the highest cross-subject mean accuracy with the lowest variability among the three γ-centric configurations (Table 2). We therefore adopt the 24-ROI high-γ power representation as the canonical spectral baseline. Among the non-spectral families, broadband line length attains the strongest standalone performance on the same pilot task (0.747±0.092), outperforming catch22 temporal descriptors and all coupling variants. Line length is accordingly retained as a second, morphology-based baseline. All subsequent feature-addition models are evaluated on top of both baselines at both ROI resolutions (24-ROI core and 50-ROI extended parcellations).

The Top-20 maps for high-γ power and line length (S2 and S5 Fig) show a shared concentration in early and ventral visual cortex (V1/V2, fusiform, inferior temporal), with LL additionally capturing broader broadband structure. To test whether each family contributes information beyond the other, we define three cross-family add-on blocks that are applied symmetrically to the two baselines. For a γ baseline, the add-ons are: (i) LL restricted to four bilateral ventral-visual ROIs (V1, V2, fusiform, inferior temporal; 8 features), probing whether local morphology in the most visually driven regions improves over γ power alone; (ii) LL residuals at all ROIs, obtained by linearly regressing LL on same-ROI γ power and concatenating the residuals, which isolates broadband shape variance not explained by γ amplitude; and (iii) LL residuals from the *K* least redundant ROIs (ranked by |corr(LL,γ)|), which concentrates the add-on on regions where LL and γ are least correlated while keeping the feature count compact. For an LL baseline, the same constructions are applied with the roles of LL and γ reversed, yielding visual-only γ, γ residuals for all ROIs, and γ residuals for the least-redundant *K* ROIs. These designs directly test cross-family complementarity under matched feature budgets and correspond to the first three rows in Tables 5 and 6.

**Table 5:** 8-class, 24-ROI models with high-γ vs. LL baseline. Mean ± SD across 16 subjects. Each row compares two models that share the same add-on feature set but differ only in the baseline: high-γ power vs. broadband LL. In the “8 visual ROIs (opposite family)” row, the add-on consists of 8 features from the opposite family (LL for the high-γ baseline and high-γ for the LL baseline).

Add-on Feature Set	high-γ Baseline	LL Baseline
	Acc. ± SD (#feats)	Acc. ± SD (#feats)
8 visual ROIs (opposite family)	0.743±0.102 (32)	0.747±0.098 (32)
residuals, all ROIs (opposite family)	0.729±0.105 (48)	0.746±0.095 (48)
residuals, top-*K* ROIs (opposite family)	0.732±0.104 (39)	0.745±0.098 (39)
phase (imag-coh, wPLI)	0.716±0.105 (56)	0.726±0.098 (72)
orthogonalized AAC	0.727±0.107 (40)	0.733±0.097 (48)
α/β PLV (feedback edges)	0.723±0.110 (40)	0.723±0.099 (72)
catch22 (short horizon, *k*/ROI)	0.725±0.103 (96)	0.738±0.100 (96)
catch22 residual	0.729±0.102 (96)	0.738±0.097 (96)

**Table 6:** 8-class, 50-ROI models with high-γ vs. LL baseline. Mean ± SD across 16 subjects. As in Table 5, each row compares two models that share the same add-on feature set but use either a high-γ or LL baseline over all 50 ROIs.

Add-on Feature Set	high-γ Baseline	LL Baseline
	Acc. ± SD (#feats)	Acc. ± SD (#feats)
8 visual ROIs (opposite family)	0.764±0.101 (58)	0.779±0.097 (58)
residuals, all ROIs (opposite family)	0.752±0.108 (100)	0.777±0.094 (100)
residuals, top-*K* ROIs (opposite family)	0.760±0.104 (65)	0.777±0.098 (65)
phase (imag-coh, wPLI)	0.734±0.117 (162)	0.748±0.111 (162)
orthogonalized AAC	0.752±0.106 (82)	0.765±0.096 (82)
α/β PLV (feedback edges)	0.736±0.110 (158)	0.748±0.106 (158)
catch22 (short horizon, *k*/ROI)	0.750±0.105 (200)	0.765±0.097 (200)
catch22 residual	0.750±0.103 (200)	0.762±0.099 (200)

The single-family screen indicates that catch22 carries useful but distributed temporal information. The full catch22 achieves an accuracy of 0.521±0.096, and the Top-20 features are dominated by short-horizon descriptors (local forecast error, short-lag autocorrelation decay, successive-difference statistics, and three-point motifs) recurring across multiple ROIs. In contrast, an unsupervised 24-dimensional PCA compression substantially degrades performance to 0.394±0.082. These patterns motivate two compact add-on designs. A “short-horizon, top-*k*/ROI” stack retains a small set (*k* = 3) of the most informative short-horizon catch22 descriptors per ROI, focusing on statistics that emerge as discriminative while keeping the feature count modest. A second, residualized variant linearly regresses each selected catch22 statistic on the corresponding baseline feature from the same ROI and uses the residuals as features. This representation isolates morphology-related variance not explained by the baseline descriptor, so any gain in accuracy can be interpreted as complementary non-oscillatory temporal structure added on top of either spectral or morphology-based baselines [27].

The single-family screen indicates that coupling features alone provide measurable but limited discriminative power in this paradigm. High-γ phase synchrony (PLV) is the most informative of the three families, whereas PAC and AAC contribute only weak, spatially diffuse effects at the single-trial level. Because PLV-type metrics are susceptible to field spread and zero-lag mixing [78], we treat coupling as a secondary descriptor and, in subsequent add-on analyses, focus on compact, connectivity blocks that are both data-guided and supported by prior work. First, we refine the phase-synchrony signal by applying imaginary coherency and the weighted phase lag index (wPLI) to visually and parietally anchored edge sets, both of which were designed to down-weight zero-lag interactions and improve sensitivity to genuine inter-areal synchrony [31,78]. Second, we include orthogonalized AAC between visual and association hubs, following evidence that amplitude-envelope correlations form stable large-scale networks when corrected for source leakage [32,65]. Finally, we include an α/β PLV (8–30 Hz) block from parietal/prefrontal to visual regions to probe feedback-related coordination, motivated by reports that α/β rhythms preferentially carry feedback influences whereas γ-band activity carries feedforward signals in visual hierarchies [2].

The combination analyses proceed with two baselines: high-γ band power as the canonical spectral descriptor and broadband line length as a complementary morphology descriptor. All add-on feature sets (cross-family power/LL blocks, catch22 variants, and coupling blocks) are evaluated on top of each baseline separately, allowing us to assess the additional information each family contributes beyond these baselines.

### Baseline models with feature-family add-ons

We now move to compact combinations on the same 8-class pilot, evaluated at both spatial resolutions: the 24-ROI core and 50-ROI extended parcellations. For each resolution, we consider two baselines: ROI high-γ power and broadband LL, and attach the candidate add-on sets (including cross-family γ/LL blocks, short-horizon and residualized *catch22*, and compact coupling blocks). A separate RF classifier is trained per participant for every baseline–add-on configuration, and performance is reported as cross-subject mean ± SD accuracy across the 16 participants (Tables 5 and 6).

#### Add-ons on the high-γ baseline.

On the high-γ power baseline, all add-on families produced higher cross-subject mean accuracies than the high-γ baseline alone on the 8-class pilot (Table 5 and Table 6). The largest mean-accuracy increases come from cross-family blocks that add LL features in ventral visual cortex. With 24 ROIs, adding LL from the 8 bilateral visual ROIs (V1, V2, fusiform, inferior temporal) raises mean accuracy to 0.743±0.102 using only 32 features; relative to the 24-ROI high-γ baseline (0.674±0.100), this improvement was supported by a paired Wilcoxon signed-rank test (*n* = 16, mean paired difference = 0.068, median paired difference = 0.073, *p* < 0.001). With 50 ROIs, the analogous visual LL block reaches 0.764±0.101 with 58 features. Residualized LL blocks (all ROIs or least-redundant *K*) also show higher cross-subject mean accuracies than the high-γ baseline but do not exceed the visual-only design, indicating that the most informative morphology is concentrated in early and ventral visual areas rather than being uniformly distributed across the cortex.

Catch22 and coupling add-ons provide smaller gains on top of γ. Short-horizon catch22 stacks (with or without residualization) reach ≈0.725−0.729 at 24 ROIs and ≈0.750 at 50 ROIs (Table 5 and 6), consistent with catch22 acting as a diffuse, low-magnitude complement to band-limited power. Compact coupling blocks, including imaginary coherency and wPLI on selected edges, orthogonalized AAC, and α/β PLV feedback edges, yield accuracies in the 0.716–0.736 range and remain below the best LL and catch22 combinations. Permutation-based feature-importances (S10 and S11 Figs) show that high-γ power in early/ventral visual cortex continues to dominate the top ranks, with visual LL features entering among the most important predictors when included, and coupling features contributing lower-rank, distributed edges.

On the high-γ baseline, the most efficient add-on is a small LL block restricted to ventral visual ROIs: it yields the highest accuracy for a given feature count at both ROI resolutions and provides a clear mechanistic interpretation as a transient/morphology complement to local high-frequency power.

#### Add-ons on the line-length baseline.

On the LL baseline, the analogous LL + 8 high-γ configuration is among the highest-performing feature combinations in our candidate set, but its mean accuracy remains numerically similar to LL alone on the 8-class pilot (0.747±0.092 vs. 0.747±0.098 at 24 ROIs). A paired Wilcoxon signed-rank test did not support an improvement for LL + 8 high-γ relative to LL-only (*n* = 16, median paired difference = 0.001, *p* = 0.528). This is consistent with the interpretation that broadband LL already captures most of the variance shared with ventral visual high-γ in this setting.

Other high-γ-based add-ons behave similarly. Residual high-γ blocks, whether taken over all ROIs or restricted to a least-redundant *K* set, yield accuracies of 0.745–0.777 across 24- and 50-ROI resolutions (Tables 5 and 6), with no clear evidence of a systematic gain over the LL-only baseline. Catch22-based add-ons achieve 0.738±0.100 at 24 ROIs and 0.762–0.765 at 50 ROIs, while coupling stacks (phase, orthogonalized AAC, α/β PLV) reach 0.723–0.748. At the level of cross-subject mean accuracy, none of these combinations shows a consistent improvement over LL alone.

Feature-importance maps clarify how the add-ons are used. For all LL-based combinations, the Top-20 permutation-importance ranks are dominated by LL features in early and ventral visual, parietal, and prefrontal ROIs, with catch22 and coupling entries appearing mainly below this Top-20 band. The LL + 8 γ stack is the only LL-based configuration in which ventral visual high-γ features consistently enter the cross-subject Top-20 at both 24 and 50 ROIs. This pattern is consistent with LL and visual high-γ reflecting overlapping aspects of the same underlying visual response: the model can distribute splits across both without a statistically supported change in overall accuracy. Permutation-importance scores for high-γ features are lower than those for LL but clearly non-zero, indicating that the classifier assigns meaningful weight to these spectral cues even though the cross-subject mean accuracy remains very similar to the LL-only baseline on this pilot.

#### Selection of final stacks.

Within the constrained set of baselines and add-on families examined, the two baselines already account for most of the attainable decoding performance on the 8-class pilot (Tables 5 and 6). Cross-family feature add-ons provide configuration-dependent gains.

On the high-γ baseline, the strongest cross-family visual add-on in the present candidate set comes from adding LL features in the four bilateral ventral visual ROIs (V1/V2, fusiform, inferior temporal). This high-γ+8 LL stack achieves the highest cross-subject mean accuracy at both ROI resolutions for only a small increase in dimensionality, and at 24 ROIs its improvement over the high-γ baseline is supported by paired testing. Permutation-based feature-importances show that, across 24 and 50 ROIs, visual LL features are the only add-on family that reliably enters the Top-20 ranks alongside dominant high-γ power (S10 and S12 Figs).

On the LL baseline, the LL + 8 high-γ configuration shows similar subject-level performance to LL alone on the pilot, with no statistically supported improvement observed in the 24-ROI analysis. LL therefore serves as a compact morphology-only reference. LL + 8 high-γ is retained because it is the only LL-based stack in which ventral visual high-γ features consistently appear in the Top-20 importance ranks at both ROI resolutions, providing a symmetric spectral add-on for comparison with high-γ+8 LL.

For the full EEG-ImageNet benchmarks (All-80, Coarse-40, Fine-40), we therefore carry forward three feature stacks: LL on all ROIs as a non-spectral baseline; high-γ power on all ROIs plus LL in the 8 ventral visual ROIs (high-γ+8 LL); and LL on all ROIs plus high-γ power in the same 8 visual ROIs (LL + 8 high-γ). Together, these stacks span a parsimonious morphology-only reference, the strongest high-γ-based cross-family visual stack identified on the pilot, and a matched LL-based cross-family visual stack retained for symmetric comparison rather than for a statistically supported gain over LL alone.

### Evaluation on full EEG-ImageNet label sets

We now evaluate the three selected stacks on the full EEG-ImageNet benchmark. For each participant, separate RF models are trained for the All-80, Coarse-40, and Fine-40 label sets at both spatial resolutions (24-ROI core and 50-ROI extended parcellations), using the same cross-validation scheme and hyperparameters as in the pilot.

All-80 results are summarized in Table 7, and Coarse-40 and Fine-40 results are reported in Table 8.

**Table 7:** Cross-subject accuracy on All-80 classes. Values are mean ± SD.

Method	No. of Features	All (80)
Original RF baseline	310	34.9±8.7
*24-ROI stacks*		
24 ROIs, LL baseline	24	38.7±11.2
24 ROIs, high-γ+8 LL	32	36.2±10.5
24 ROIs, LL + 8 high-γ	32	39.0±10.8
*50-ROI stacks*		
50 ROIs, LL baseline	50	42.2±11.1
50 ROIs, high-γ+8 LL	58	40.7±10.5
50 ROIs, LL + 8 high-γ	58	42.1±11.1

**Table 8:** Cross-subject accuracy on Coarse-40 and Fine-40 categories. Values are mean ± SD.

Method	No. of Features	Coarse (40)	Fine (40)
Original RF baseline	310	45.4±10.5	72.9±7.2
*24-ROI stacks*			
24 ROIs, LL baseline	24	49.2±10.9	78.0±6.6
24 ROIs, high-γ+8 LL	32	47.1±11.5	75.8±7.0
24 ROIs, LL + 8 high-γ	32	49.3±11.1	78.2±6.5
*50-ROI stacks*			
50 ROIs, LL baseline	50	52.5±11.3	80.0±6.5
50 ROIs, high-γ+8 LL	58	51.3±11.7	77.8±7.2
50 ROIs, LL + 8 high-γ	58	52.4±11.1	80.1±6.5

The EEG-ImageNet sensor-space baseline uses differential-entropy features across 62 channels and five frequency bands (310 features), achieving 34.89% (All-80), 45.35% (Coarse-40), and 72.88% (Fine-40) with a Random Forest classifier [12]. In contrast, our source-space ROI stacks achieve higher reported mean accuracy than this previously reported benchmark while using substantially fewer features. With the 24-ROI core parcellation, the LL-only baseline (24 features; ≈92% reduction relative to 310) reaches 38.7% (All-80), 49.2% (Coarse-40), and 78.0% (Fine-40), while the two ventral-visual cross-family variants achieve similar subject-aggregated mean accuracy (36.2–39.0% All-80; 47.1–49.3% Coarse-40; 75.8–78.2% Fine-40). With the 50-ROI extended parcellation, the LL-only baseline (50 features; ≈84% reduction) provides the strongest overall results, reaching 42.2% (All-80), 52.5% (Coarse-40), and 80.0% (Fine-40), with LL + 8γ showing numerically similar subject-aggregated mean accuracies (42.1%, 52.4%, and 80.1%, respectively).

Overall, compact ROI-targeted stacks compare favorably, at the level of reported mean accuracy, with the high-dimensional channel×band baseline while using far fewer features. For the LL-only baseline, increasing spatial resolution from 24 to 50 ROIs yielded statistically supported improvements across all three label sets. Relative to the 24-ROI model, the 50-ROI model achieved higher subject-level accuracy on All-80 (0.422±0.111 vs. 0.387±0.112), Coarse-40 (0.525±0.113 vs. 0.492±0.109), and Fine-40 (0.800±0.065 vs. 0.780±0.066), with paired Wilcoxon signed-rank tests supporting these gains in all three settings (all two-sided *p*-values $=3.1\times 10^{-5}$; mean paired improvements = 0.035, 0.033, and 0.020, respectively). By contrast, adding high-γ power as a small anatomically constrained block to the LL baseline does not show a clear numerical advantage on the full benchmark: γ+8 LL remains below LL, whereas LL + 8 high-γ remains very similar to LL at the subject-aggregated level. Accordingly, LL-only is adopted as the primary baseline, with LL + 8 high-γ retained as a matched spectral add-on for symmetric cross-family comparison.

To better understand the relatively large cross-subject SDs in Tables 7 and 8, we examined participant-level LL-only accuracies across the All-80, Coarse-40, and Fine-40 settings at both ROI resolutions (S13 Fig). Two consistent patterns emerge. First, the lower tail is not distributed uniformly across participants, but is driven primarily by a small subset of consistently low-performing participants, particularly Subjects 10 and 12, whose accuracies remain comparatively low across all three label sets and both ROI resolutions. This pattern suggests that a meaningful portion of the observed heterogeneity is subject-specific rather than tied to any single label granularity. Second, the improvement from 24 to 50 ROIs is broadly distributed across participants in all three settings, indicating that the gain from finer ROI granularity is not driven by only one or two high-performing subjects. Instead, the extended parcellation appears to recover additional discriminative variance in a relatively consistent manner across subjects, even though absolute decoding levels remain heterogeneous.

To characterize which ROIs drive LL-only decoding, we first summarize the highest-ranked ROI features for the 24-ROI and 50-ROI LL-only models on the All-80 label set in S14 Fig. Detailed across-ROI importance distributions are provided in S15 and S16 Figs. The same core ROIs repeatedly appear among the most informative features across label sets (All/Coarse/Fine), reflecting a posterior–anterior mix that includes early visual cortex (V1/V2/cuneus; and OccipitalPole at higher resolution), ventral temporal regions (IT and parahippocampal cortex), and frontal regions (dlPFC and superior frontal). This pattern indicates that LL-only performance is supported by a distributed set of sources rather than a single dominant ROI. The 50-ROI setting redistributes importance into more specific parcels: the 24-ROI representation places noticeable weight on broader posterior midline/parietal regions (e.g., precuneus/PCC and SPL/IPL), whereas the 50-ROI representation highlights finer parcels available only at higher resolution (e.g., OccipitalPole and STS), alongside additional attention/oculomotor-related parcels (e.g., IPS and FEF proxies) and category-relevant proxy regions (e.g., MT/EBA/LOC; most evident in the Fine split, not shown). Thus, the 50-ROI parcellation does not merely add features but changes which named regions the model relies on, effectively replacing coarse aggregates with finer-grained parcels.

## Discussion

In this paper, we showed that accurate visual object decoding on EEG-ImageNet does not require high-dimensional sensor-space feature sets. After source localization, a minimal LL-only representation compares favorably, at the level of reported mean accuracy, with the previously reported channel×band differential-entropy Random Forest benchmark while using ∼80–90% fewer features. Across the All-80, Coarse-40, and Fine-40 label sets, this LL-only representation is the most parsimonious top performer at both spatial resolutions (24-ROI core and 50-ROI extended). Accordingly, the value of the present analysis is not only that it attains strong reported accuracy on this benchmark, but that it shows much of the decodable information can be recovered with a compact source-space representation that preserves explicit anatomical attribution and a substantially lower feature budget.

Under 0.5 s single-trial RSVP windows, these results indicate that a lightweight time-domain summary of each ROI time course is sufficient to capture most decodable variance. Line length is sensitive to both amplitude changes and rapid fluctuations while remaining computationally simple and low-latency [54,79]. In this regime, LL provides a strong default baseline for cross-subject decoding and an attractive feature for practical deployments where tight compute budgets and reproducibility matter.

High-γ remains important to evaluate because high-frequency activity has been associated with local population activity and visual representations, yet it is also susceptible to non-neural contamination in non-invasive recordings [15,52,53]. The lack of systematic gain from adding ROI-restricted high-γ suggests that, for this dataset and validation setting, performance is dominated by time-domain structure already captured by LL. More broadly, recent decoding work has emphasized that waveform-shape features can provide information not reducible to power alone [80]; here, LL offers a simple instantiation of this idea without introducing additional model complexity.

The feature-importance profiles further indicate that LL-based decoding is supported by a stable, distributed set of ROIs rather than a single dominant source (S14, S15, and S16 Figs). Across label sets, the highest-ranked LL features consistently involve a posterior–anterior mix spanning early visual cortex, ventral temporal ROIs, and frontal ROIs, suggesting that discriminative time-domain structure is shared across multiple systems. The statistically supported gains of the 50-ROI LL-only model further suggest that the observed improvement reflects benefits of increased spatial resolution beyond additional dimensionality alone. Rather than simply expanding the feature set, the extended parcellation appears to shift attribution from broad posterior aggregates toward finer higher-resolution parcels while capturing additional discriminative variance across subjects. At the same time, the finer anatomical attribution afforded by the 50-ROI representation should be interpreted more cautiously under the present template-based source-modeling and representative-vertex summarization pipeline, because smaller and more irregular parcels may be more sensitive to local misregistration, source leakage, and within-parcel heterogeneity than broader aggregates.

An additional methodological boundary concerns the rule used to summarize each ROI as a single time series. In this study, each parcel was represented by one centroid-nearest dipole waveform rather than by within-ROI averaging, sign-aware averaging, or PCA/SVD-based summaries. This choice was intentional: it preserves a transparent one-parcel-to-one-feature mapping, keeps dimensionality low, and limits additional mixing or cancellation that can arise when multiple source points with heterogeneous local orientations are collapsed into a single parcel average. At the same time, alternative summary rules are reasonable and may be more robust in parcels with greater internal heterogeneity, local misregistration, or residual leakage, because they can average across multiple vertices or align dominant within-ROI variance before extracting a representative waveform [48]. The present anatomical attribution and decoding results should therefore be interpreted as conditional on a compact representative-vertex summary under a template-based source-modeling pipeline [47]. A useful extension would be a controlled comparison of representative-vertex, sign-aware averaging, and PCA/SVD-based parcel summaries within the same decoding framework.

Participant-level accuracies further indicate that the relatively large cross-subject SDs reflect structured heterogeneity rather than uniformly broad dispersion. A small subset of participants, particularly Subjects 10 and 12, occupied the lower tail across all three label sets and both ROI resolutions, whereas most remaining participants fell within a narrower accuracy range. This pattern is consistent with a subject-specific component in decoding difficulty that is not tied only to label granularity. Under the present source-modeling pipeline, one plausible contributor is inter-subject variation in the fidelity of source reconstruction when individual anatomy and digitized electrode locations are unavailable [13,34]. Additional contributors may include differences in single-trial RSVP response strength, attentional engagement, and residual post-cleaning signal-to-noise ratio. Notably, the accuracy gain from 24 to 50 ROIs was distributed across most participants rather than being confined to the highest-performing subjects, suggesting that the finer parcellation may capture additional discriminative variance in a reasonably general manner even though absolute accuracy remains heterogeneous across individuals.

Beyond LL and band power, coupling (PLV, imaginary coherency, wPLI, orthogonalized AAC) and catch22 morphology stacks provided only small and inconsistent improvements under the strict single-trial, cross-participant regime. Two factors likely contribute: (i) short windows reduce the reliability of connectivity estimates, and leakage-robust metrics can further attenuate weak interactions; and (ii) RSVP responses exhibit inter-subject variability in latency and waveform detail, limiting the incremental separability captured by fixed-window morphology descriptors once amplitude- and shape-sensitive summaries are included [27,32]. Larger gains from coupling and morphology may emerge with longer effective windows (e.g., pseudo-trials), latency alignment, or individualized ROI/band definitions [81,82].

From an applications standpoint, the proposed source-space ROI representation is compatible with real-time deployment. In the present study, source estimates were computed offline, but the same workflow can be implemented online once the subject-specific forward model and inverse operator are prepared. The anatomy-dependent steps, including BEM construction, forward computation, and inverse estimation, constitute a one-time per-subject calibration, after which incoming EEG can be projected to cortical sources at millisecond cadence. Online processing then reduces to applying the precomputed inverse to buffered data, extracting ROI time courses, and computing lightweight per-ROI features such as line length [23,24,83].

Open-source toolchains explicitly support this streaming pattern. MNE-CPP’s MNE Scan performs real-time MNE source localization on evoked and single-trial data and can recompute the forward solution when head movement is detected [22,84]. Reported end-to-end latencies for real-time source estimation are compatible with closed-loop operation (e.g., clustered real-time beamforming achieving ∼15–16 ms total delay for 10 ms windows), implying negligible overhead relative to typical BCI decision windows [85]. Related work in EEGLAB/BCILAB similarly demonstrates near real-time source mapping on live EEG streams [86]. Because the representation compresses data into a small set of ROI “virtual sensors” and uses computationally trivial features, the incremental cost after source projection is minimal. When streaming source estimation is unavailable, subject-specific spatial filters derived from the inverse operator can approximate ROI time series in sensor space while preserving anatomical targeting.

Several factors limit the scope of these conclusions. The results are established within the EEG-ImageNet RSVP paradigm and time-locked 0.5 s single-trial epochs, and therefore primarily generalize to rapid visual categorization under similar timing and task demands.

A further limitation concerns the interpretation of results obtained on EEG-ImageNet in light of prior discussion around block-design visual EEG datasets. In the present study, the evaluation code did not mix training and test data within a participant, and models were evaluated using shuffled stratified 5-fold cross-validation. However, this does not eliminate the broader benchmark-level caveat that block-structured stimulus presentation, in which samples from the same class appear consecutively rather than in fully randomized order, can introduce temporal dependencies, reduce effective variability, and increase redundancy across nearby trials unrelated to stimulus identity [87]. Accordingly, the present findings should be interpreted as showing that compact, anatomically interpretable source-space features achieve strong reported performance under this benchmark and evaluation protocol, rather than as resolving the broader methodological debate surrounding block-design visual EEG datasets. More broadly, the central contribution of this work lies less in maximizing benchmark scores and more in demonstrating that careful construction of compact source-space feature representations can recover substantial discriminative information under realistic data constraints. Future datasets with more randomized stimulus presentation, reduced temporal redundancy, and broader trial diversity may provide an even stronger testbed and could further improve achievable performance.

Source reconstruction relied on a template head model (fsaverage) and a standard sensor montage rather than subject-specific anatomy and digitized electrode locations. This choice can increase spatial blurring and leakage, so ROI-level findings should be interpreted at a coarse systems level (e.g., occipital, ventral temporal, or frontoparietal groupings) rather than as precise focal loci.

Finally, feature-importance profiles are descriptive and model-dependent; permutation importance reflects contributions within the trained Random Forest and can be influenced by correlated features and inter-subject variability, and should not be interpreted as a causal measure of regional necessity.

## Conclusion

This study evaluated compact source-space feature stacks for rapid visual object decoding on EEG-ImageNet. Cortical activity was summarized as ROI time courses and compared using consistent cross-validation across the All-80, Coarse-40, and Fine-40 label sets.

Across both spatial resolutions (24-ROI and 50-ROI), the LL-only baseline provided the strongest accuracy–parsimony trade-off. A single line length feature per ROI showed higher reported mean accuracy than the reported higher-dimensional sensor-space differential-entropy Random Forest baseline while substantially reducing the feature count. This finding supports an accuracy–parsimony trade-off rather than a purely performance-driven objective. Adding a small, anatomically constrained high-γ block yielded near-tied performance rather than a systematic improvement. Coupling and catch22 morphology features provided limited incremental benefit under strict single-trial 0.5 s windows. PCA compression of catch22 reduced accuracy, indicating that discriminative temporal structure is not captured by a very low-dimensional projection.

These results support a practical design choice for EEG-based visual decoders and noninvasive BCIs. A low-dimensional ROI-level representation with lightweight time-domain features can achieve strong decoding accuracy with transparent anatomical attribution and low computational cost. Because the inverse operator can be calibrated once per participant and applied to streaming data, the resulting pipeline is compatible with real-time use.

Future work should test generalization beyond RSVP to longer or naturalistic viewing conditions and evaluate source estimates with individual head models and digitized electrode locations. Additional gains may emerge from participant-specific tuning of analysis windows, ROI definitions, and feature parameters, particularly for coordination and morphology-based features.

## Supporting information

S1 FigTop-20 aggregated feature importances for five band powers with γ (30–120 Hz) in the 24-ROI setting.(PNG)

S2 FigTop-20 aggregated feature importances for γ (70–150 Hz) band power in the 24-ROI setting.(PNG)

S3 FigTop-20 aggregated feature importances for γ (30–120 Hz) band power in the 24-ROI setting.(PNG)

S4 Fig Top-20 cross-subject feature importances for catch22 features in the 24-ROI setting. (PNG)

S5 Fig Top-20 cross-subject feature importances for line-length (LL) features in the 24-ROI setting. (PNG)

S6 FigTop-20 cross-subject feature importances for γ-band phase-locking value (PLV) with amplitude weighting in the 24-ROI setting.(PNG)

S7 Fig Top-20 cross-subject feature importances for the concatenated amplitude-weighted coupling stack (PLV + PAC + AAC) in the 24-ROI setting. (PNG)

S8 FigTop-20 cross-subject feature importances for α→high-γ phase–amplitude coupling (PAC) in the 24-ROI setting.(PNG)

S9 FigTop-20 cross-subject feature importances for β–high-γ amplitude–amplitude coupling (AAC) in the 24-ROI setting.(PNG)

S10 FigTop-20 aggregated feature importances for γ (70–150 Hz) band power with 8 visual LL features.Bars show mean permutation importance across participants; error bars indicate ±SD.(PNG)

S11 FigTop-20 common feature importances across subjects for high-γ (70–150 Hz) band power with visual–parietal/prefrontal cortex (PFC) orthogonalized AAC features.(PNG)

S12 FigTop-20 cross-subject feature importances for the LL+γ stack (visual ROIs only) at 50 ROIs.(PNG)

S13 Fig Participant-level LL-only accuracy by ROI resolution across the All-80, Coarse-40, and Fine-40 label sets. Bars show per-subject mean 5-fold accuracy for the 24-ROI and 50-ROI LL-only models in each label set. Across all three settings, the lower tail is driven primarily by a small subset of consistently low-performing participants, particularly Subjects 10 and 12, while the improvement from 24 to 50 ROIs is broadly distributed across subjects.
(PNG)

S14 Fig Top-ranked ROIs for LL-only decoding at two spatial resolutions on the All-80 label set. Panel A shows the Top-10 ROI features for the 24-ROI LL-only model, ranked by mean permutation importance across participants. Panel B shows the corresponding Top-10 ROI features for the 50-ROI LL-only model. Across both parcellations, the highest-ranked ROIs form a distributed posterior–anterior pattern that includes early visual, ventral temporal, and frontal systems. Error bars indicate ±SD across participants.
(PNG)

S15 Fig Across-participant feature importance for the LL-only baseline on the All-80 label set (24 ROIs). Bars show mean permutation-based feature importance across participants; error bars indicate ±SD.
(PNG)

S16 Fig Across-participant feature importance for the LL-only baseline on the All-80 label set (50 ROIs). Bars show mean permutation-based feature importance across participants; error bars indicate ±SD.
(PNG)

S1 Appendix Preprocessing and model parameters. Detailed denoising, preprocessing, classifier, and training parameters used in the experiments.
(PDF)

S1 Table ROI organization by functional system. Functional ROI names are shown with the corresponding FreeSurfer Destrieux (aparc.a2009s) atlas labels used for representative dipole selection.
(PDF)
