# Supplementary figures and images for: Decoding visual object recognition from EEG signals
Source: PLoS One. 2026 Jun 24;21(6):e0351872. doi: 10.1371/journal.pone.0351872 (PMC13293449; doi:10.1371/journal.pone.0351872)

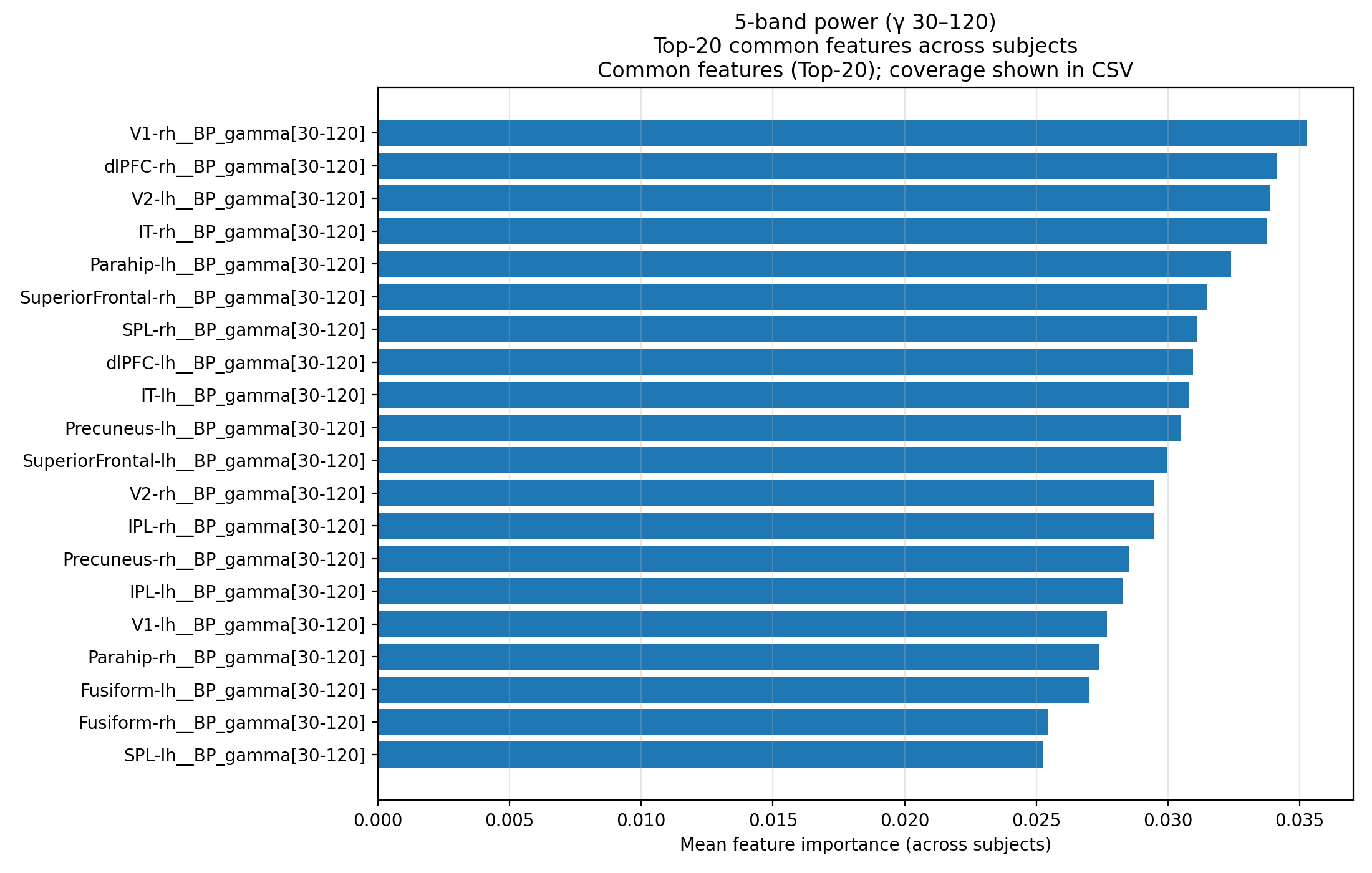

Supplement: S1 Fig — (PNG) [file pone.0351872.s001.png]

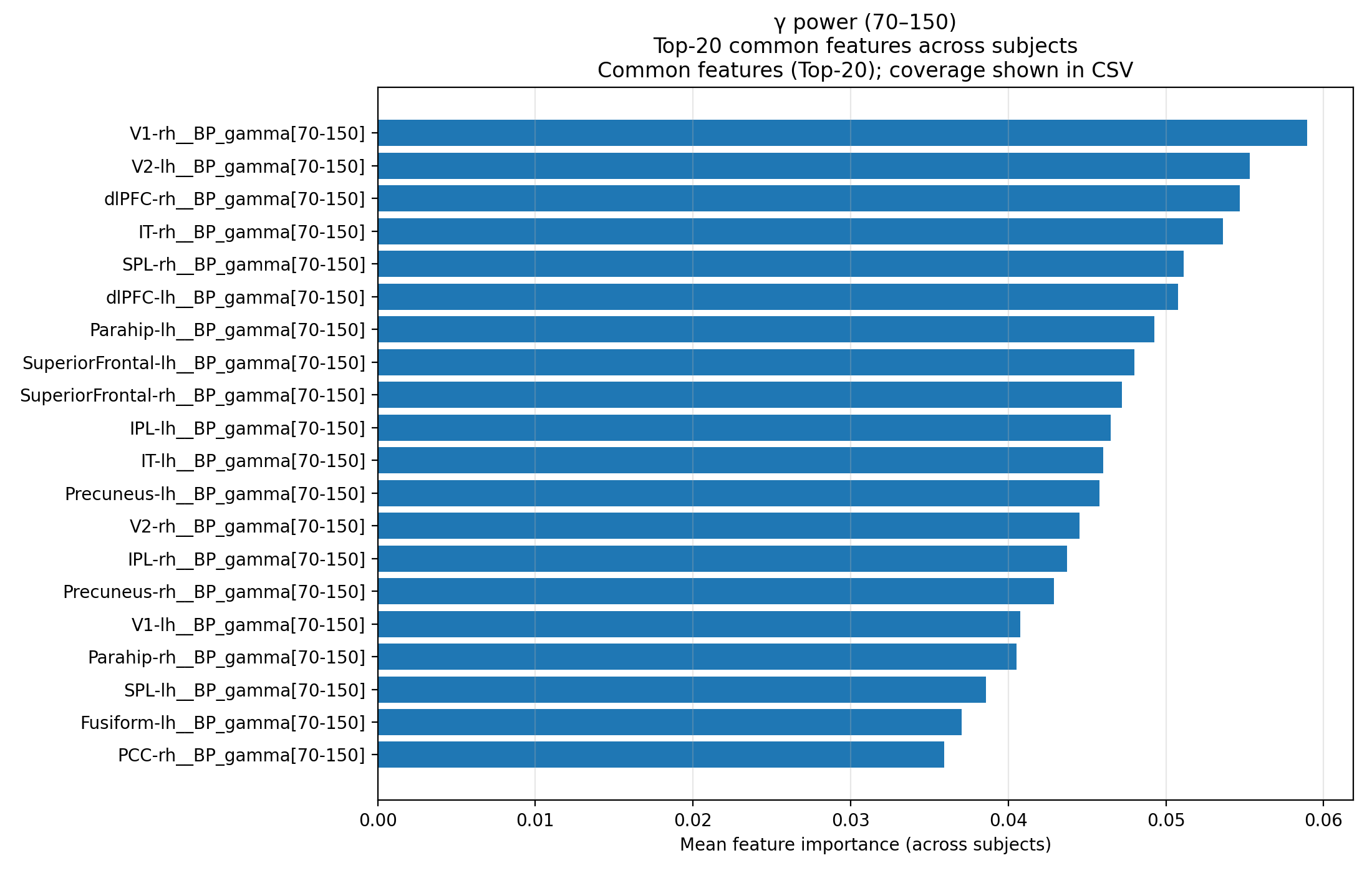

Supplement: S2 Fig — (PNG) [file pone.0351872.s002.png]

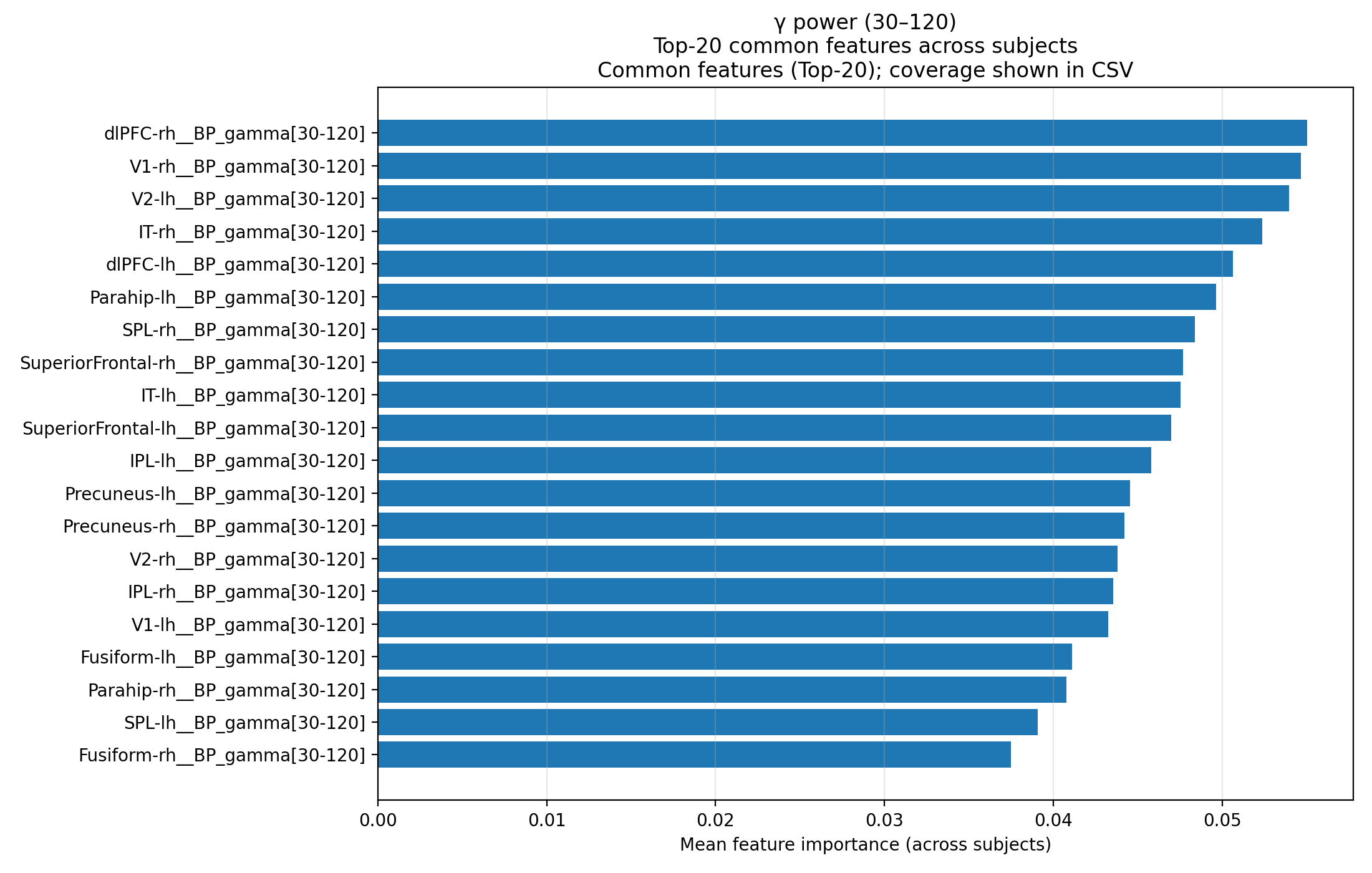

Supplement: S3 Fig — (PNG) [file pone.0351872.s003.png]

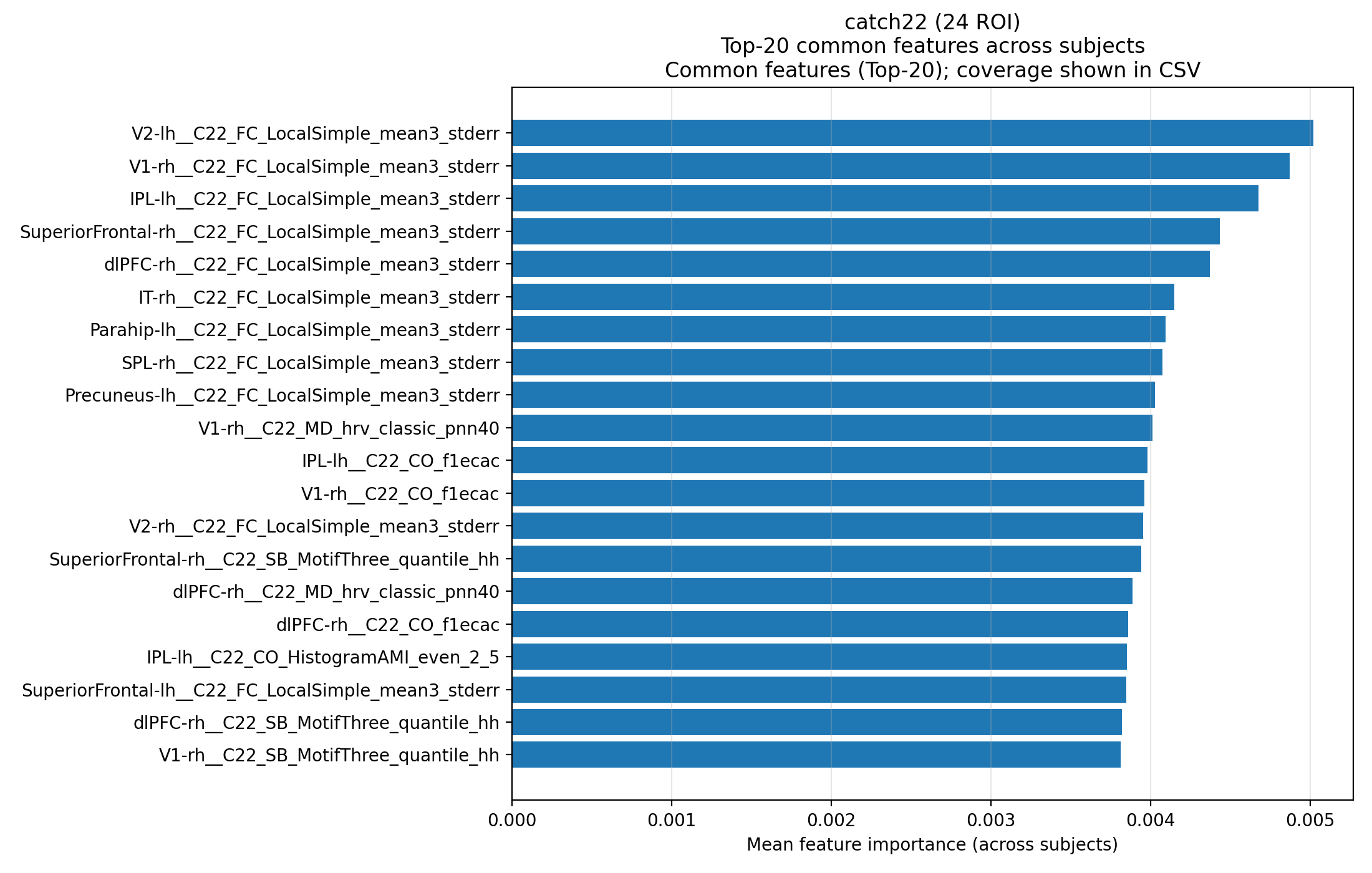

Supplement: S4 Fig — (PNG) [file pone.0351872.s004.png]

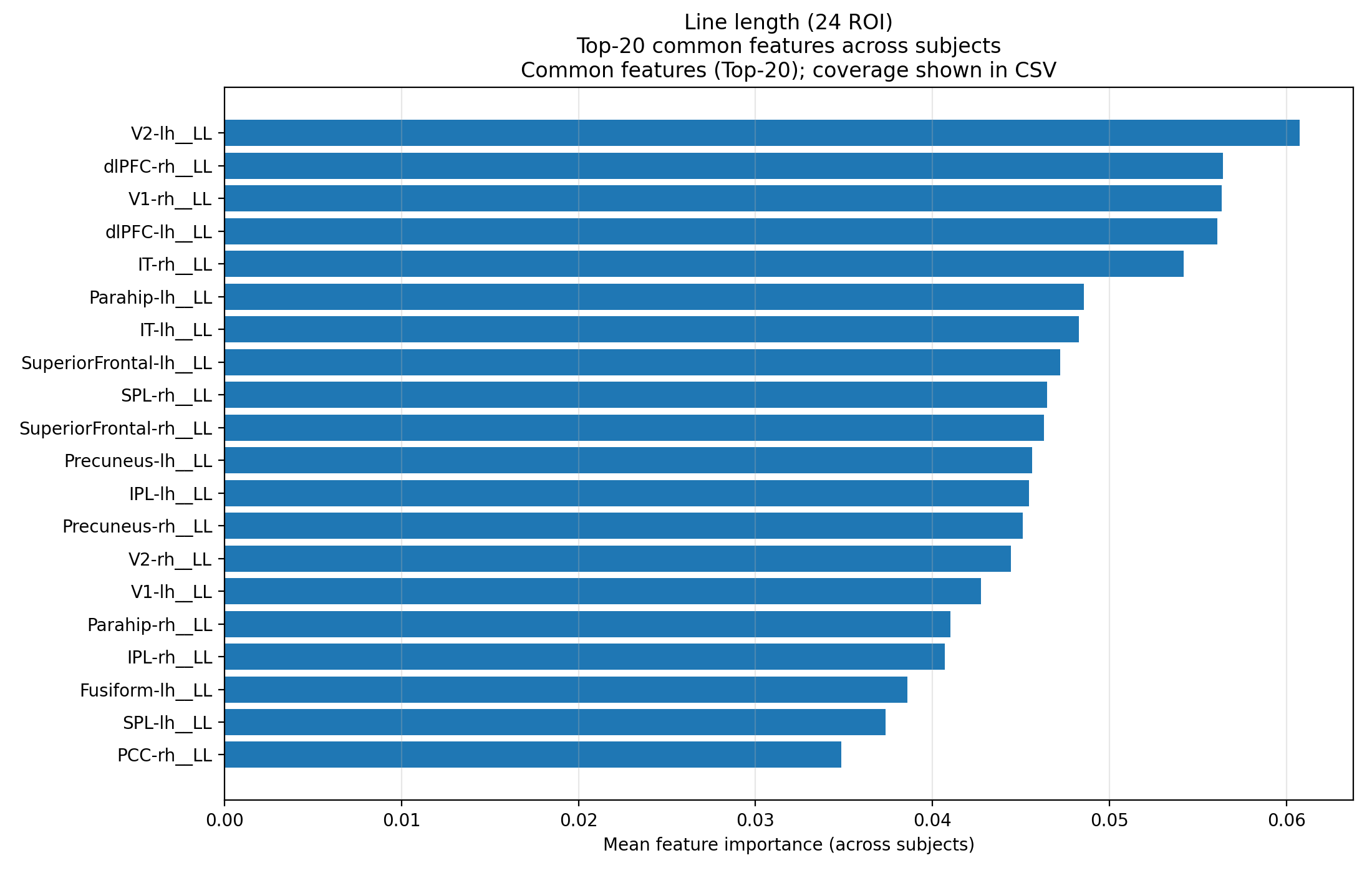

Supplement: S5 Fig — (PNG) [file pone.0351872.s005.png]

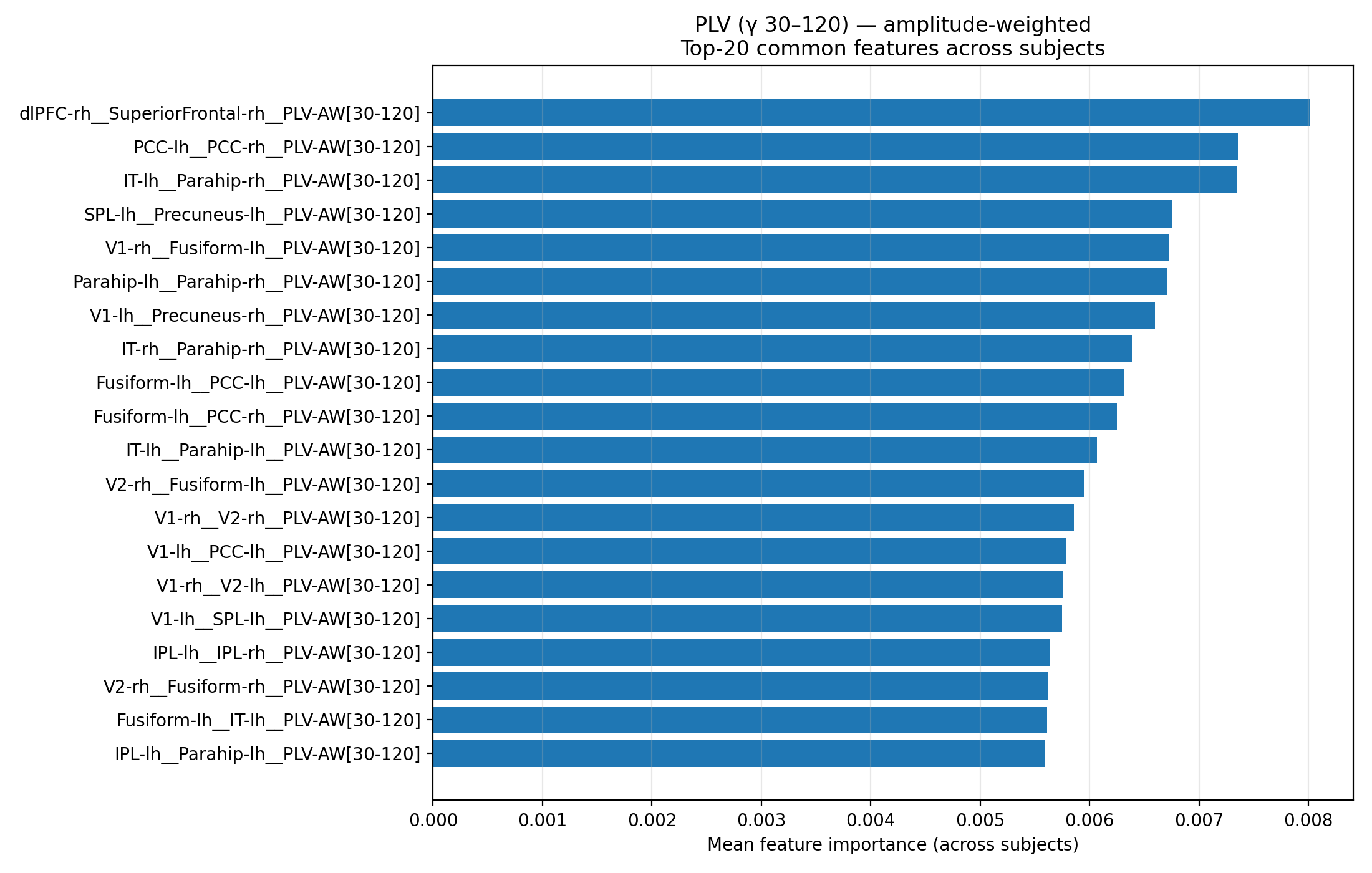

Supplement: S6 Fig — (PNG) [file pone.0351872.s006.png]

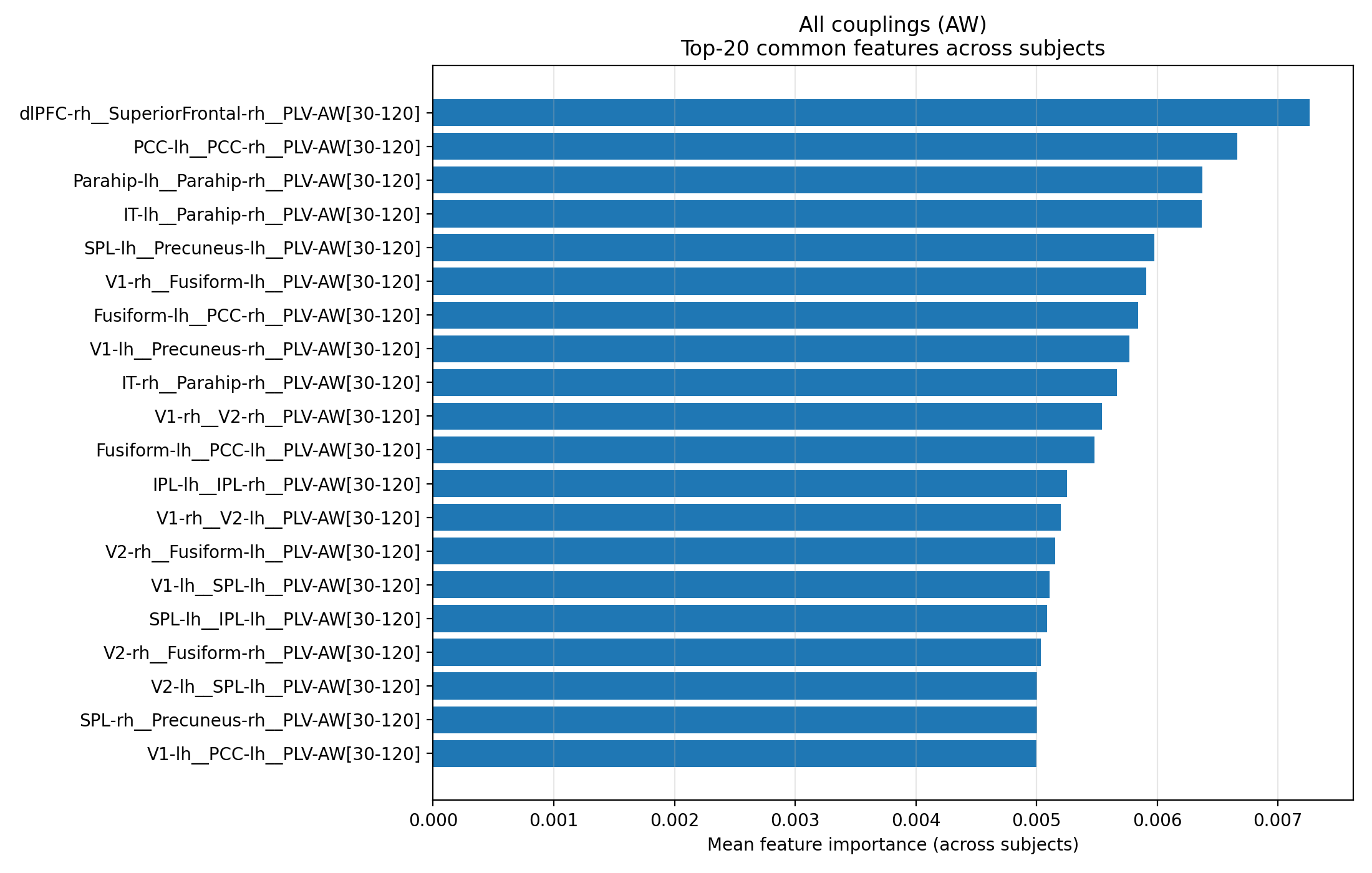

Supplement: S7 Fig — (PNG) [file pone.0351872.s007.png]

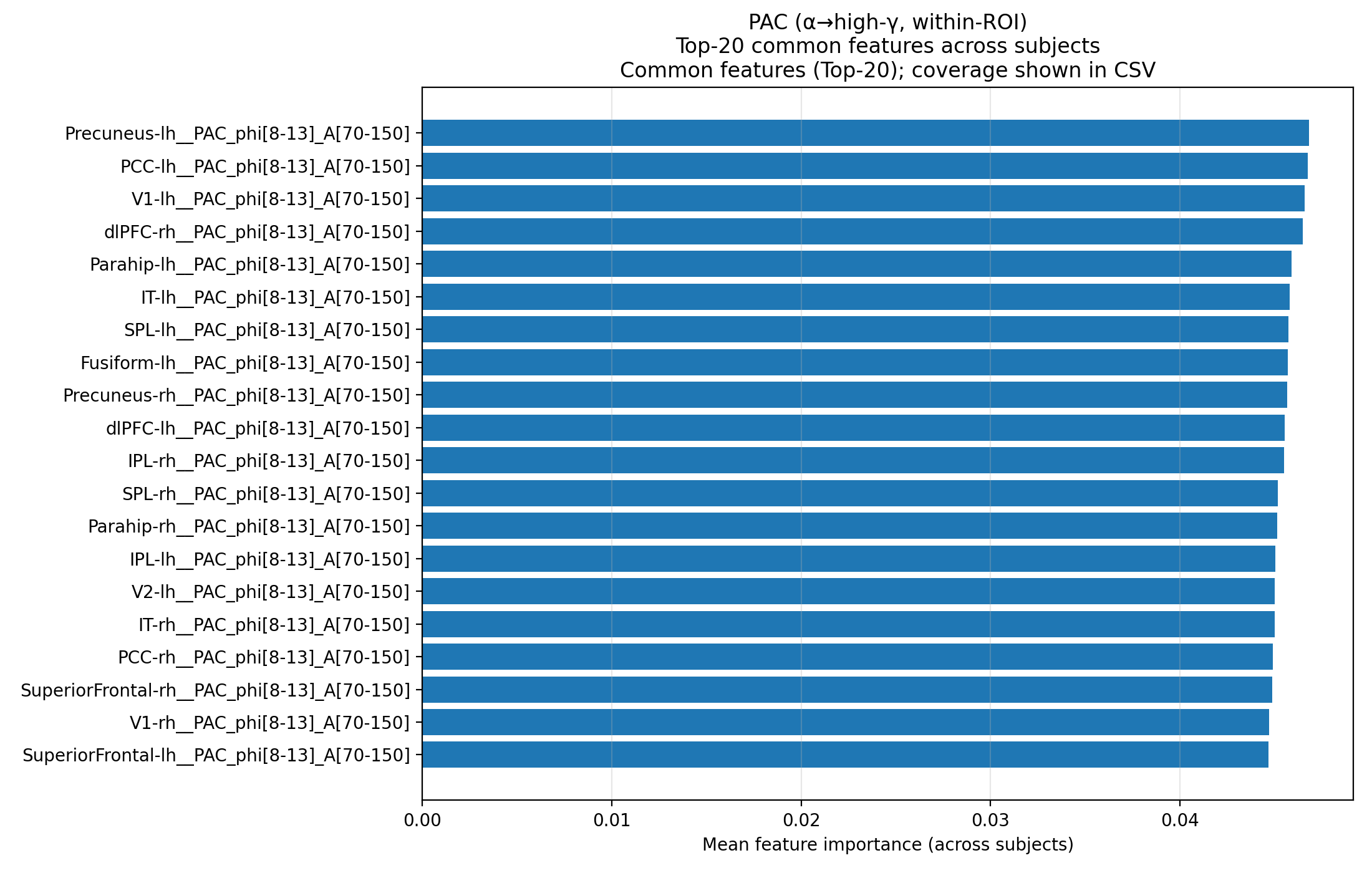

Supplement: S8 Fig — (PNG) [file pone.0351872.s008.png]

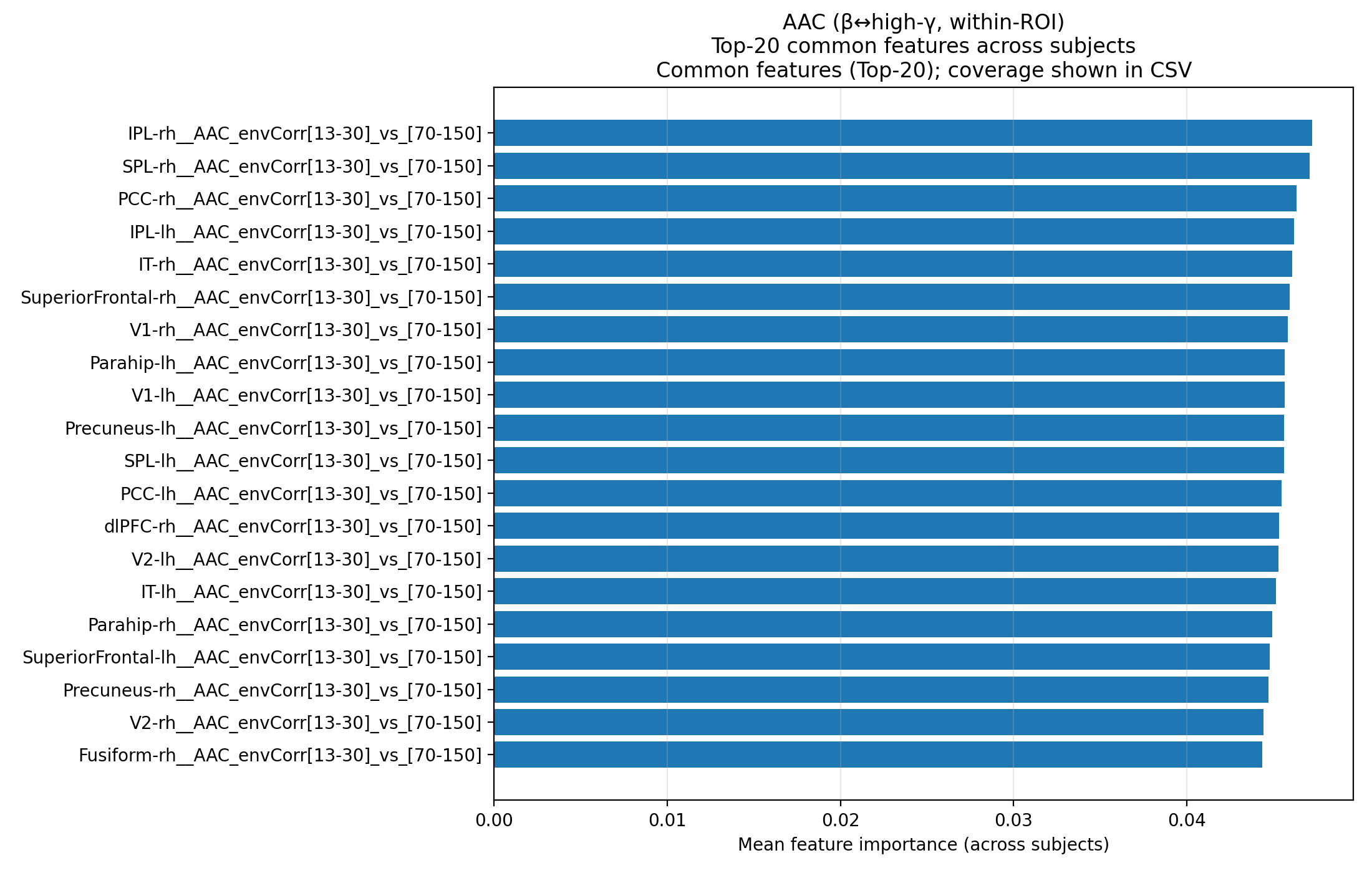

Supplement: S9 Fig — (PNG) [file pone.0351872.s009.png]

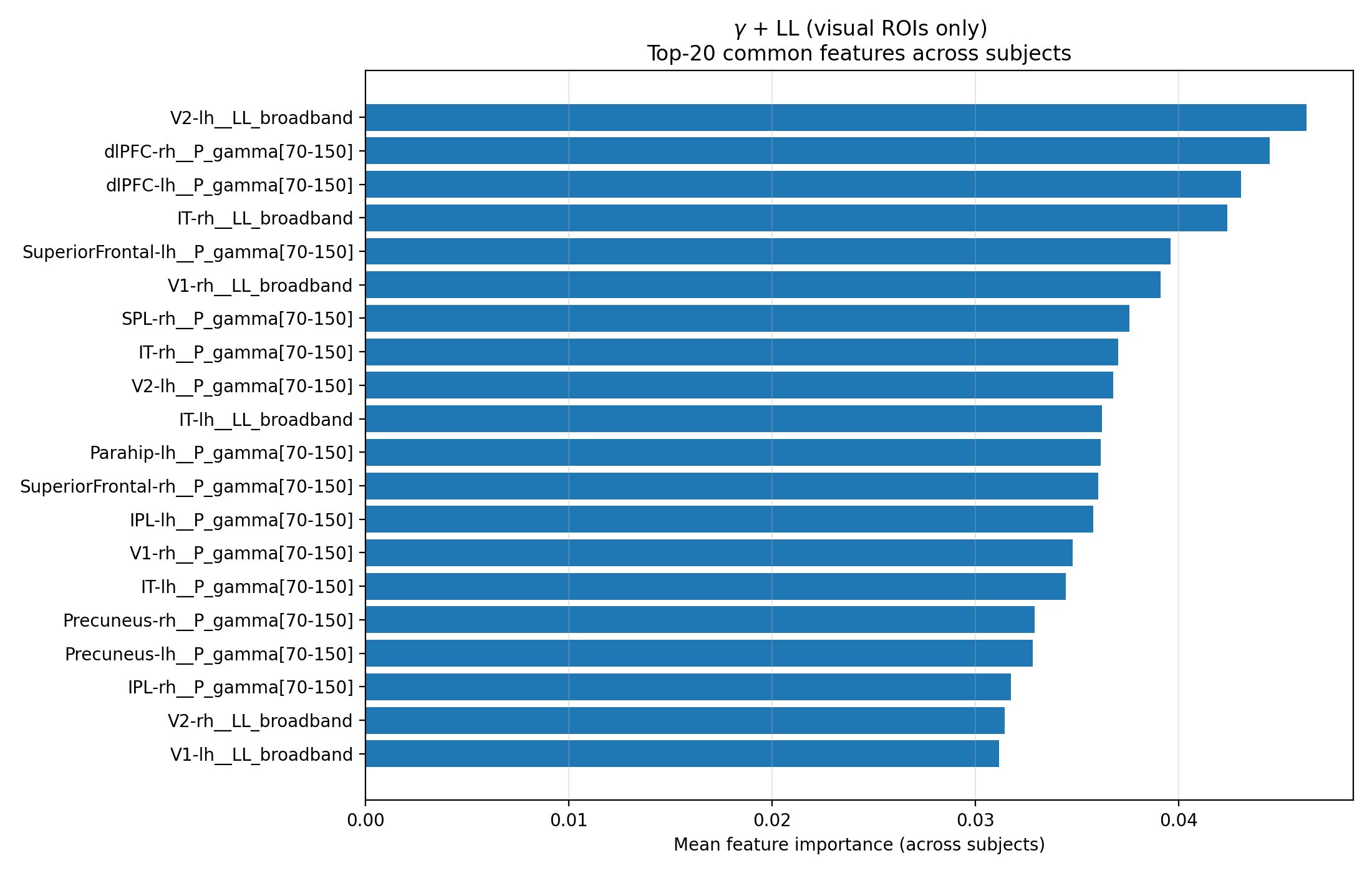

Supplement: S10 Fig — Bars show mean permutation importance across participants; error bars indicate ±SD. (PNG) [file pone.0351872.s010.png]

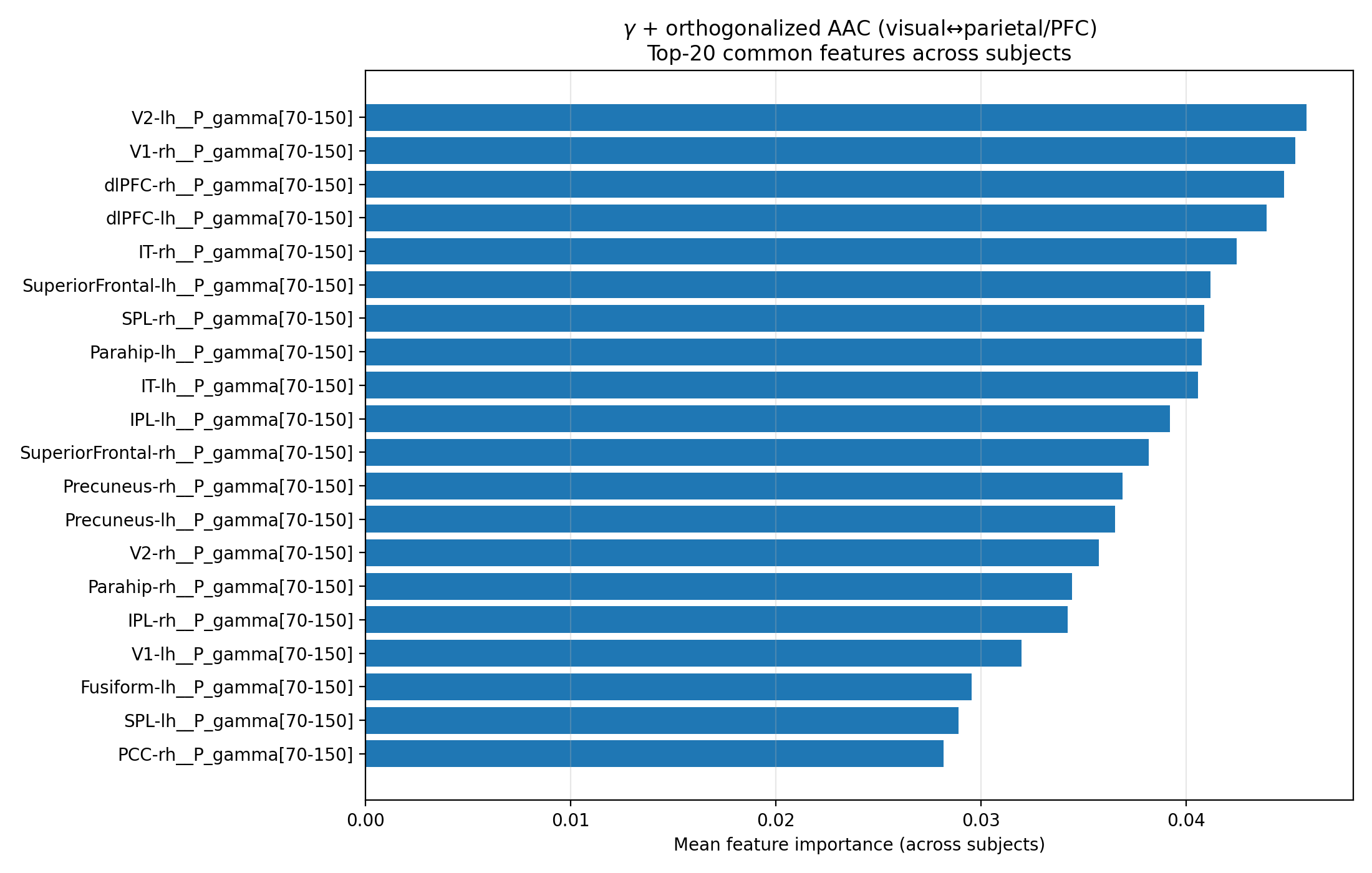

Supplement: S11 Fig — (PNG) [file pone.0351872.s011.png]

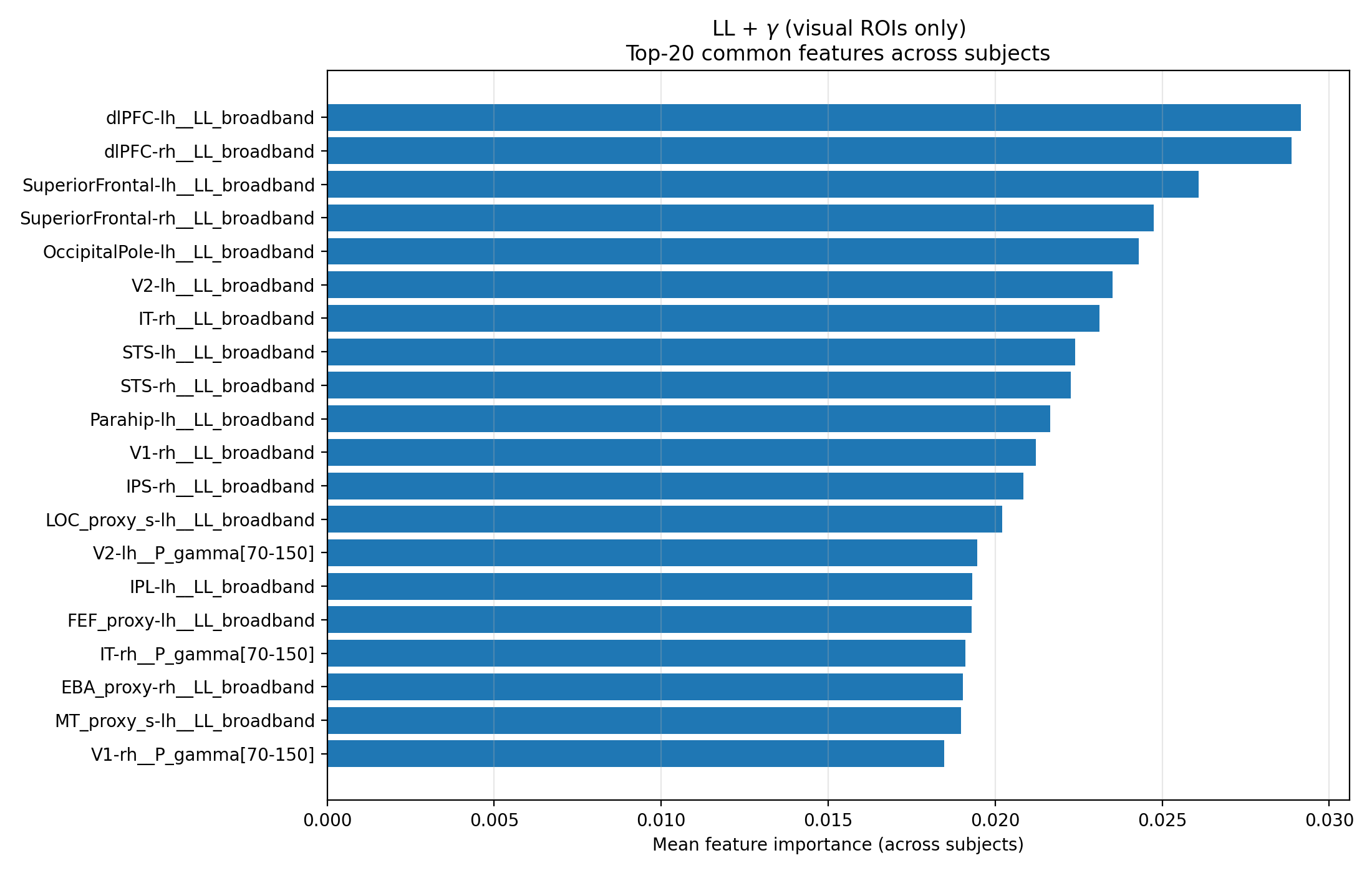

Supplement: S12 Fig — (PNG) [file pone.0351872.s012.png]

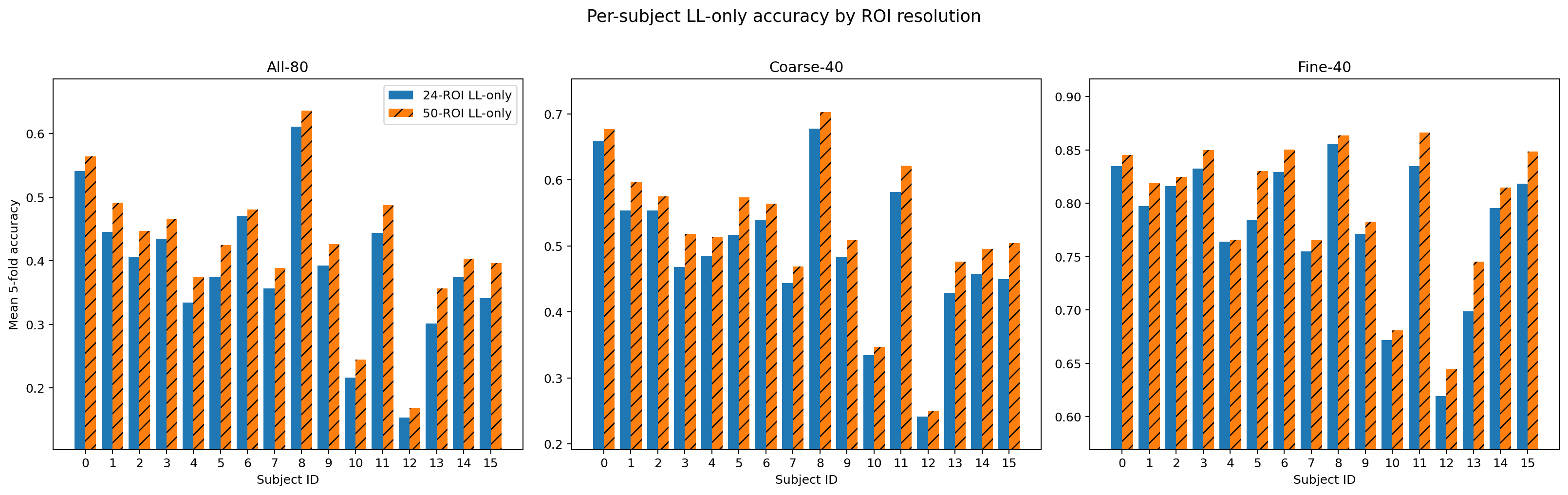

Supplement: S13 Fig — Bars show per-subject mean 5-fold accuracy for the 24-ROI and 50-ROI LL-only models in each label set. Across all three settings, the lower tail is driven primarily by a small subset of consistently low-performing participants, particularly Subjects 10 and 12, while the improvement from 24 to 50 ROIs is broadly distributed across subjects. (PNG) [file pone.0351872.s013.png]

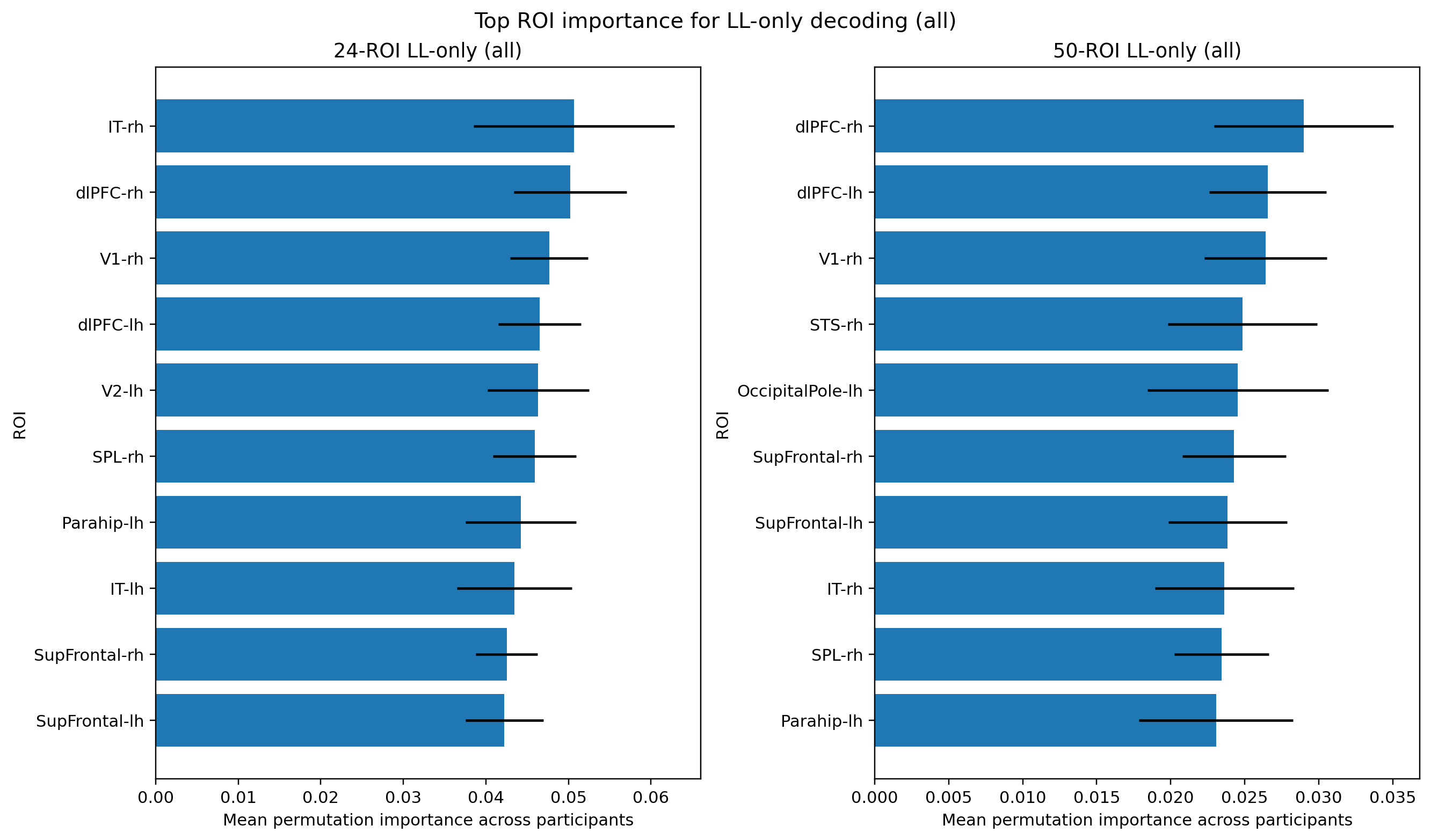

Supplement: S14 Fig — Panel A shows the Top-10 ROI features for the 24-ROI LL-only model, ranked by mean permutation importance across participants. Panel B shows the corresponding Top-10 ROI features for the 50-ROI LL-only model. Across both parcellations, the highest-ranked ROIs form a distributed posterior–anterior pattern that includes early visual, ventral temporal, and frontal systems. Error bars indicate ±SD across participants. (PNG) [file pone.0351872.s014.png]

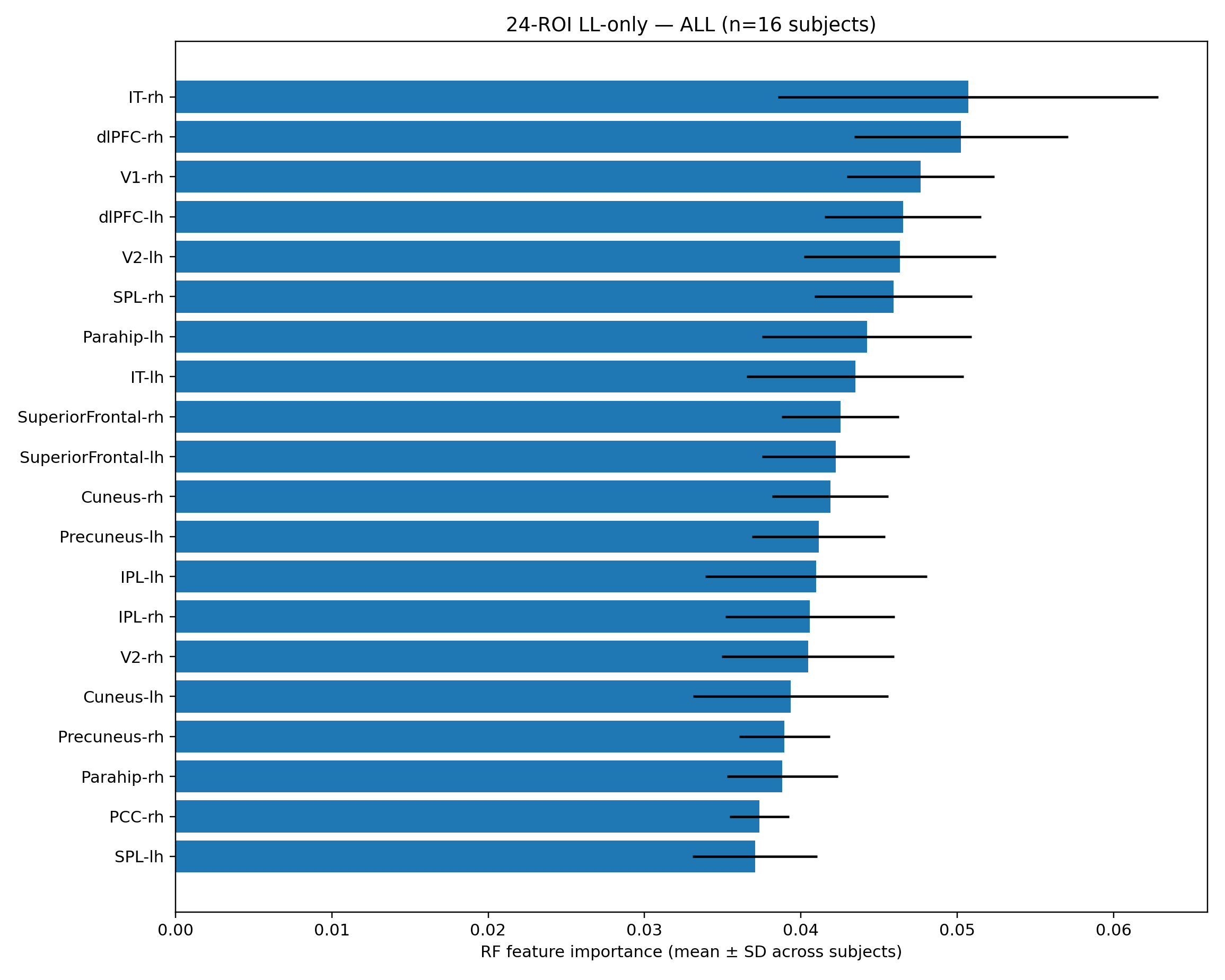

Supplement: S15 Fig — Bars show mean permutation-based feature importance across participants; error bars indicate ±SD. (PNG) [file pone.0351872.s015.png]

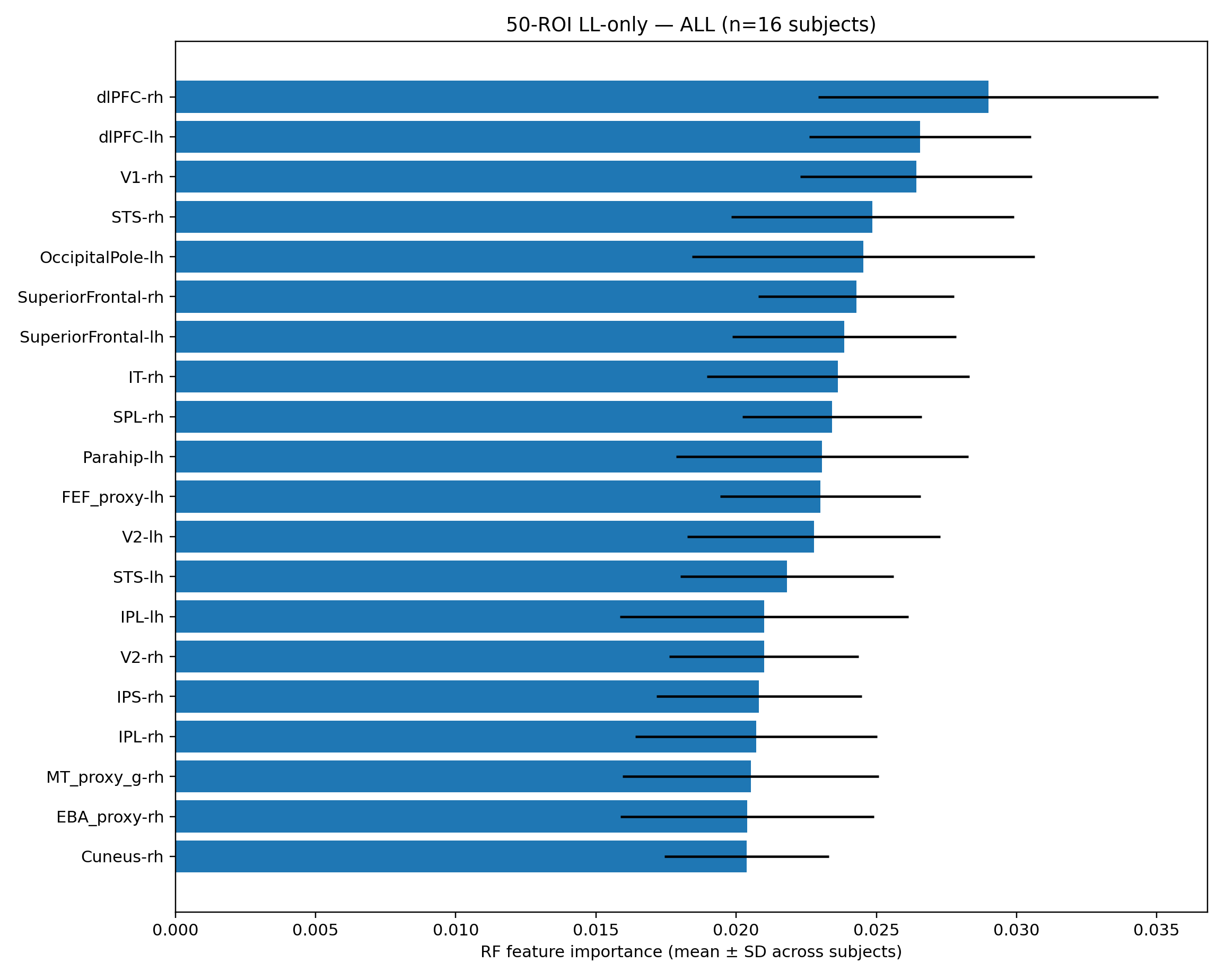

Supplement: S16 Fig — Bars show mean permutation-based feature importance across participants; error bars indicate ±SD. (PNG) [file pone.0351872.s016.png]
