# Supplementary material for: Decoding visual object recognition from EEG signals
Source: PLoS One. 2026 Jun 24;21(6):e0351872. doi: 10.1371/journal.pone.0351872 (PMC13293449; doi:10.1371/journal.pone.0351872)
Supplement: S1 Appendix — Detailed denoising, preprocessing, classifier, and training parameters used in the experiments. (PDF) [file pone.0351872.s017.pdf]

S1 Appendix. Preprocessing and model parameters.

Denoising parameters

Filter: FIR, 0.5–150 Hz, zero-phase; notch: 50/100/150 Hz.

ICA: FastICA, $n = 62$; reject if $r(\text{EOG}) > 0.3$ or ICLabel muscle/eye > 0.8 or EMG-$z > 3$.

Bad channel: flat > 1 s, p2p $> 150\,\mu$V, corr < 0.6.

Epoch reject: p2p $> 150\,\mu$V or EMG-$z > 3$.

Reference: common average.

Classifier and training

All unspecified scikit-learn parameters were left at their library defaults.

For classifier selection on the 8-class pilot, candidate classifiers were evaluated using the same 24-ROI high-γ (70–150 Hz) band-power representation, the same subject set, and the same stratified 5-fold cross-validation splits. Within each subject, class labels were integer-encoded, and a single `StratifiedKFold` partition (`n_splits=5`, `shuffle=True`, `random_state=42`) was generated and then reused across all candidate classifiers to ensure matched train/test folds.

- **Random Forest:** Random Forest (scikit-learn): 500 trees, Gini criterion, unlimited depth, `max_features="sqrt"`, `class_weight="balanced"`, bootstrap enabled, `random_state=42`, and all CPUs used.
- **Linear SVM:** Linear SVM (scikit-learn `LinearSVC`): features were standardized by `StandardScaler`; `C=1.0`, `class_weight="balanced"`, `max_iter=10000`.
- **ℓ_2-regularized logistic regression:** ℓ_2-regularized logistic regression (scikit-learn `LogisticRegression`): features were standardized by `StandardScaler`; `penalty="l2"`, `C=1.0`, `solver="lbfgs"`, `max_iter=1000`, `class_weight="balanced"`.
- **Ridge classifier:** Ridge classifier (scikit-learn `RidgeClassifier`): features were standardized by `StandardScaler`; `alpha=1.0`, `class_weight="balanced"`, `random_state=42`.
- **K-nearest neighbors:** K-nearest neighbors (scikit-learn `KNeighborsClassifier`): features were standardized by `StandardScaler`; `n_neighbors=5`, `weights="distance"`, `metric="minkowski"`, `p=2` (Euclidean distance).

Hyperparameters were set a priori from the 8-class pilot and held constant across all subjects and tasks.
