# Supplementary material for: Decoding visual object recognition from EEG signals
Source: PLoS One. 2026 Jun 24;21(6):e0351872. doi: 10.1371/journal.pone.0351872 (PMC13293449; doi:10.1371/journal.pone.0351872)
Supplement: S1 Table — Functional ROI names are shown with the corresponding FreeSurfer Destrieux (aparc.a2009s) atlas labels used for representative dipole selection. (PDF) [file pone.0351872.s018.pdf]

S1 Table. ROI organization by functional system. Functional ROI names are shown with the corresponding FreeSurfer Destrieux (aparc.a2009s) atlas labels used for representative dipole selection.

Functional System	24-ROI Core (Destrieux)	Additional in 50-ROI (Destrieux)
Visual (early/mid)	V1 (S_calcarine)<br>V2 (G_oc-temp_med-Lingual)<br>Cuneus (G_cuneus)	OccipitalPole (Pole_occipital)<br>V3V4_proxy (G_occipital_middle)<br>V3V4ventral_proxy (S_oc-temp_med_and_Lingual)
Ventral temporal/object	Fusiform (G_oc-temp_lat-fusifor)<br>IT (G_temporal_inf)<br>Parahip (G_oc-temp_med-Parahip)	LOC_proxy_g (G_occipital_middle)<br>LOC_proxy_s (S_oc-temp_lat)<br>OFA_proxy (G_occipital_middle)<br>EBA_proxy (G_temporal_middle)<br>PPA_proxy (S_oc-temp_med_and_Lingual)
Lateral temporal	—<br>—<br>—	MT_proxy_s (S_oc-temp_lat)<br>MT_proxy_g (G_temporal_middle)<br>STS (S_temporal_sup)
Parietal/dorsal attention	SPL (G_parietal_sup)<br>IPL (G_pariet_inf-Supramar)<br>Precuneus (G_precuneus)	IPS (S_intrapariet_and_P_trans)<br>—<br>—
Frontal control/oculomotor	dlPFC (G_front_middle)<br>SuperiorFrontal (G_front_sup)	FEF_proxy (G_precentral)<br>—
Medial/default mode	PCC (G_cingul-Post-dorsal)	—

Note: All listed ROIs are bilateral. The 24-ROI core set corresponds to 12 functional ROI keys across left and right hemispheres. The extended 50-ROI set consists of the 24-ROI core plus 13 additional functional ROI keys, each represented bilaterally. Representative dipoles were selected using FreeSurfer aparc.a2009s, corresponding to the Destrieux atlas. The MedialTemporal entry may appear in the ROI-selection dictionary, but it is not counted as part of the downstream 24-ROI core or extended 50-ROI feature sets.
